# The Timescale of Emergence and Spread of Turnip Mosaic Potyvirus

**DOI:** 10.1038/s41598-017-01934-7

**Published:** 2017-06-26

**Authors:** Ryosuke Yasaka, Hirofumi Fukagawa, Mutsumi Ikematsu, Hiroko Soda, Savas Korkmaz, Alireza Golnaraghi, Nikolaos Katis, Simon Y. W. Ho, Adrian J. Gibbs, Kazusato Ohshima

**Affiliations:** 1 Laboratory of Plant Virology, Department of Applied Biological Sciences, Faculty of Agriculture, Saga University, 1-banchi, Honjo-machi, Saga 840-8502 Japan; 2 The United Graduate School of Agricultural Sciences, Kagoshima University, 1-21-24, Kagoshima, 890-0065 Japan; 3 Department of Plant Protection, Faculty of Agriculture, University of Canakkale Onsekiz Mart, Canakkale, Turkey; 4 Department of Plant Protection, College of Agriculture and Natural Resources, Science and Research Branch, Islamic Azad University, Tehran, P.O. Box 14515-775 Iran; 5 Plant Pathology Laboratory, Faculty of Agriculture, Aristotle University of Thessaloniki, Thessaloniki, 540 06 Greece; 6 School of Life and Environmental Sciences, University of Sydney, Sydney, NSW 2006 Australia; 7 Emeritus Faculty, Australian National University, Canberra, ACT 2601 Australia

## Abstract

Plant viruses have important global impacts on crops, and identifying their centre and date of emergence is important for planning control measures. Turnip mosaic virus (TuMV) is a member of the genus *Potyvirus* in the family *Potyviridae* and is a major worldwide pathogen of brassica crops. For two decades, we have collected TuMV isolates, mostly from brassicas, in Turkey and neighbouring countries. This region is thought to be the centre of emergence of this virus. We determined the genomic sequences of 179 of these isolates and used these to estimate the timescale of the spread of this virus. Our Bayesian coalescent analyses used synonymous sites from a total of 417 novel and published whole-genome sequences. We conclude that TuMV probably originated from a virus of wild orchids in Germany and, while adapting to wild and domestic brassicas, spread via Southern Europe to Asia Minor no more than 700 years ago. The population of basal-B group TuMVs in Asia Minor is older than all other populations of this virus, including a newly discovered population in Iran. The timescale of the spread of TuMV correlates well with the establishment of agriculture in these countries.

## Introduction

Identifying the centre and date of emergence of plant viruses is important for planning control measures^1–4^. There have been such studies of plant viruses with single- and double-stranded DNA genomes, including begomoviruses and mastreviruses in the family *Geminiviridae* ^5, 6^ and cauliflower mosaic virus (CaMV) in the family *Caulimoviridae* ^7^. Similar analyses have been conducted for plant viruses with RNA genomes, such as cucumber mosaic virus (CMV) in the family *Bromoviridae* ^8^, and turnip mosaic virus (TuMV)^9–11^ and potato virus Y^12^ in the family *Potyviridae*. Genetic data for such studies are scarce for most viruses, except orthomyxoviruses and lentiviruses, and most have been done using partial genome sequences or using a single gene. In contrast, studies of BK^13^, influenza^14^ and Ebola^15^ viruses have been carried out using around 150 whole-genome sequences. For plant viruses, the largest study using whole-genome sequences has probably been 353 isolates of maize streak virus strain A (genome length; ~2700 nucleotides) in the family *Geminiviridae* ^16^.

One of the largest genera of plant RNA viruses is *Potyvirus*. It contains 90% of the species of the family *Potyviridae* ^17^. Potyviruses infect a wide range of monocotyledonous and dicotyledonous plant species^4^. They are spread by aphids in a non-persistent manner, and also in seed and infected living plant materials. They have flexuous filamentous particles 700–750 nm long, each of which contains a single copy of the genome. The genome is a single-stranded, positive-sense RNA molecule of approximately 10,000 nucleotides (nt). It has one major open reading frame (ORF) that is translated into one large polyprotein and with a small overlapping ORF^18^. The polyprotein is autocatalytically hydrolysed into at least ten proteins^4, 17^.

In the potyvirus phylogenetic tree, TuMV clusters with narcissus, scallion and yam viruses to form the TuMV phylogenetic group^4^. TuMV, from the genus *Potyvirus*, is one of the best-studied plant-infecting RNA viruses in terms of its evolution. TuMV damages most domestic brassica crops in modern agriculture. These plants were developed from wild brassica plants by plant breeders during the expansion of agriculture. Previous studies have shown that this virus originated from wild orchids in Europe^10^ and then spread among species of wild and domestic Brassicaceae plants, from the Mediterranean region, including South-east Europe, the Middle East and Central Asia^9, 19, 20^, to other parts of the world including East Asia^21–23^, Oceania^11^ and the Americas. There are two reports of TuMV from Middle Eastern countries^24, 25^. However, these studies reported only two whole-genome sequences, leaving considerable uncertainty about the population structure and diversity of the virus in the region.

In this study, we collected 179 TuMV isolates in Turkey, Greece and Iran over two decades, mostly from brassica hosts, and determined their genome sequences. This region is thought to be the centre of emergence and spread of this virus, and the region in which it adapted to agricultural crops. We estimated the evolutionary rate and timescale of this virus using synonymous sites^8^ and inferred its phylodynamic history using a combined data set of 417 novel and published genome sequences. These analyses reveal the present geographical structure of TuMV populations in and around the centre of TuMV emergence. Our study possibly represents the largest evolutionary study of an RNA plant virus, set in the context of the agricultural development of its hosts.

## Results

### Sample collection, virus isolation and pathogenicity

**Figure 1:**
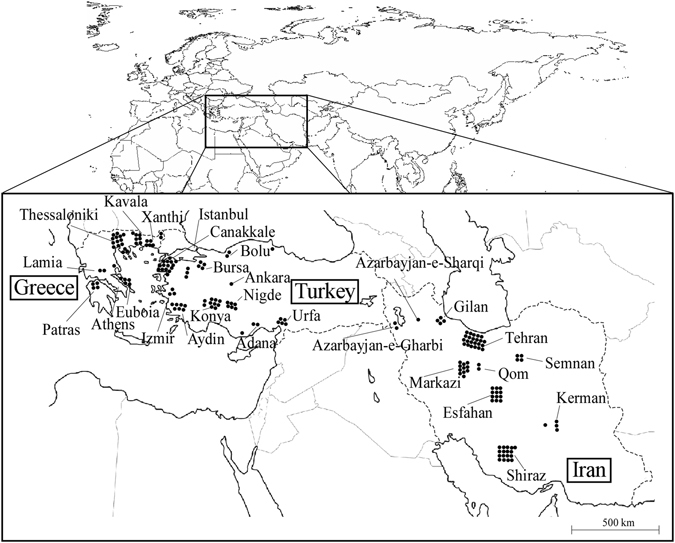
Map showing the provenance of the turnip mosaic virus isolates from Greece, Turkey and Iran. Dots on the map correspond to the isolates listed in Supplementary Table S1 (http://www.freemap.jp/about_use_map.html).

A total of 179 TuMV isolates were collected from agricultural crops and wild plants: 43 in Greece, 77 in Iran and 59 in Turkey (Fig. 1 and Supplementary Table S1). All of the Greek, Iranian and Turkish isolates infected *Brassica juncea* cv. Hakarashina and *Brassica rapa* cv. Hakatasuwari plants. However, few infected *Brassica oleracea* var. capitata cvs. Ryozan 2-go and Shinsei. Many did not infect Japanese radish (*Raphanus sativus* cvs. Akimasari and Taibyo-soubutori), but infected Chinese radish (*R. sativus* cv. Everest). Many Greek isolates did not infect radish; most of these isolates were of the [B] host-infecting type. Only a few infected radishes, perhaps because few radishes are grown in Greece (N. Katis, personal observation). In fact, we were not able to find diseased radish in Greece, and 32 out of 43 (74%) Greek isolates were [B] host-infecting type, eight were [B(R)] and none was [BR]. It was noticeable that in Turkey, which is a neighbour of Greece, we were able to find both radish crops and wild radish. Only 26 out of 59 (44%) Turkish isolates were [B] host-infecting type, 27 were [B(R)] and five were [BR]. The Iranian population was similar: 24 out of 77 (31%) were [B] host-infecting type, 39 were [B(R)] and 13 were [BR].

### Molecular characteristics and recombination analyses

We analysed the 179 sequences reported here, along with 238 whole-genome TuMV sequences obtained from online sequence databases. The publicly available data included three sequences from Greek isolates and two each from Iran and Turkey^19, 24, 25^. The 179 newly sequenced genomes had lengths of 9792–9798 nt (excluding 5′-end 35 nt primer sequences). The regions encoding the protein 1 (P1), helper-component proteinase protein (HC-Pro), protein 3 (P3), pretty interesting *Potyviridae* ORF (PIPO), 6 kDa 1 protein, cylindrical inclusion protein, 6 kDa 2 protein, genome-linked viral protein (VPg), nuclear inclusion a-proteinase protein (NIa-Pro), nuclear inclusion b protein (NIb) and coat protein (CP) had respective lengths of 1086, 1374, 1065, 177, 156, 1932–1935, 159, 573–576, 729, 1551 and 864–867 nt. The 3′ non-coding regions (NCRs) were 207–209 nt in length. All of the motifs reported for different potyvirus-encoded proteins were found.

**Figure 2:**
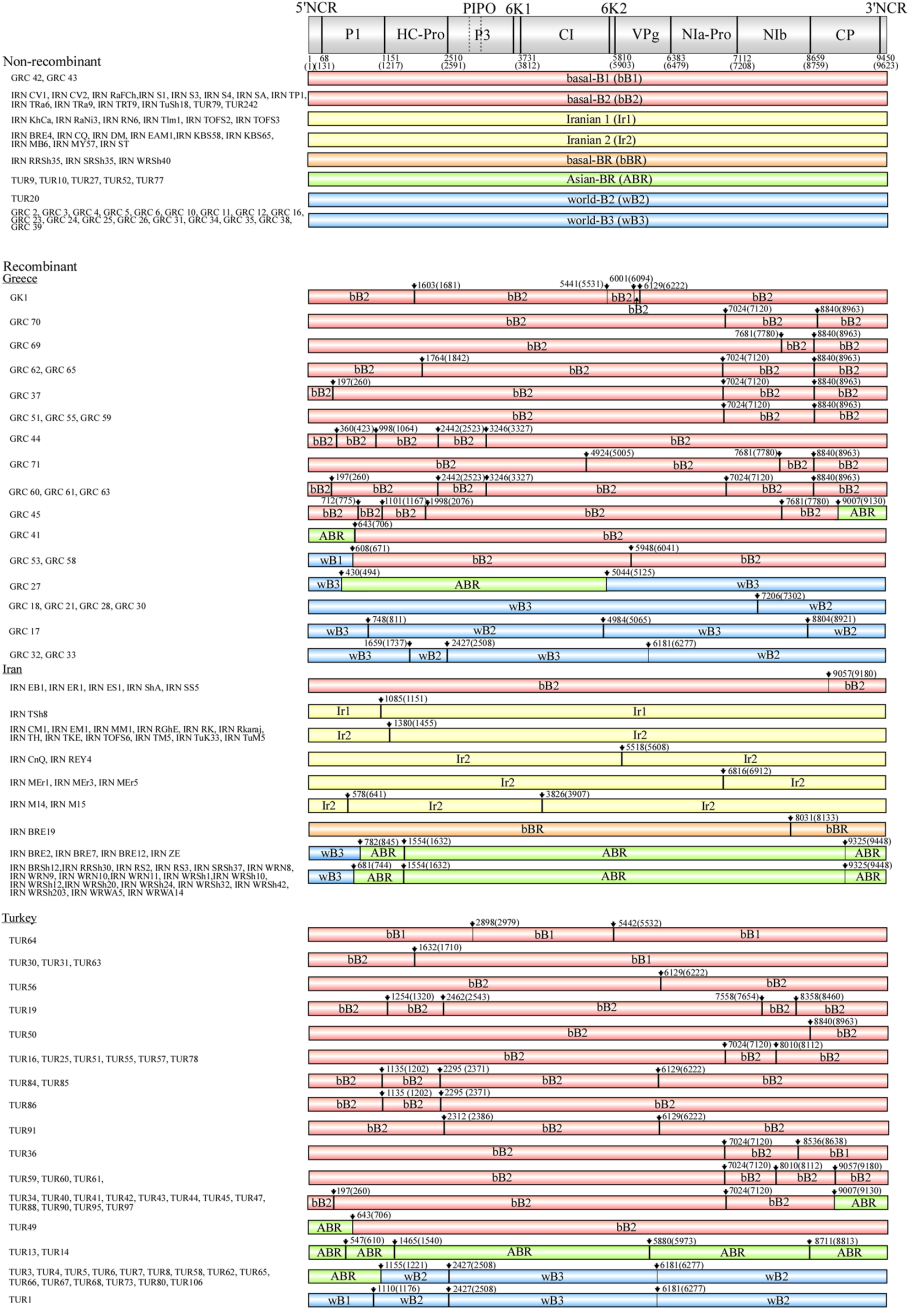
Recombination map of turnip mosaic virus genomes of the isolates from Greece, Iran and Turkey. The estimated nucleotide positions of the recombination sites and those in parentheses are shown relative to the 5′ end of the genome using the numbering of the aligned sequences used in the present study and the UK 1 isolate (Jenner *et al*.^57^). Vertical arrows and lines show estimated recombination sites (listed in Supplementary Table S2). The clear (bold line) and tentative (thin line) recombination sites identified in the present study are listed separately.

Twenty-one unequivocal recombination sites were found in the genomes of 186 Greek, Iranian and Turkish isolates (Fig. 2 and Supplementary Table S2). Only one recombination type pattern, seen in a GRC 27 isolate genome with world-B3 x Asian-BR parents, was found in an earlier study^19^. Therefore, 40 novel recombination type patterns were found among the sequences from these three countries. The commonest recombination patterns in Greek genomes were intralineage recombinants of basal-B or world-B parents. In the Iranian population, most recombinants were intralineage and had Iranian subgroup parents, but the interlineage recombinants of world-B and Asian-BR parents were also widely distributed. In Turkey, most recombinants were intralineage recombinants and had basal-B or world-B parents, as in the Greek population; however, the recombination patterns differed between the two countries.

### Phylogenetic analyses

A phylogenetic network was inferred using Neighbor-Net^26^ from the concatenated 5′ NCR, polyprotein and 3′ NCR sequences (Supplementary Fig. S1). The isolates from Greece fell into the ‘basal-B group and recombinants’ and ‘world-B group and recombinants’ clusters. The isolates from Turkey fell into ‘basal-B group and recombinants’, ‘Asian-BR group and recombinants’ and ‘world-B group and recombinant’ clusters. The isolates from Iran fell into several clusters, not only the ‘Iranian group and recombinant’ cluster, but also ‘basal-B group and recombinants’ and ‘Asian-BR group and recombinants’ clusters. Therefore, all of the isolates from these three countries fell into the ‘basal-B group and recombinants’ cluster and clustered with Italian isolates. None of the isolates from Greece, Iran or Turkey clustered with the ‘Orchis group’.

**Figure 3:**
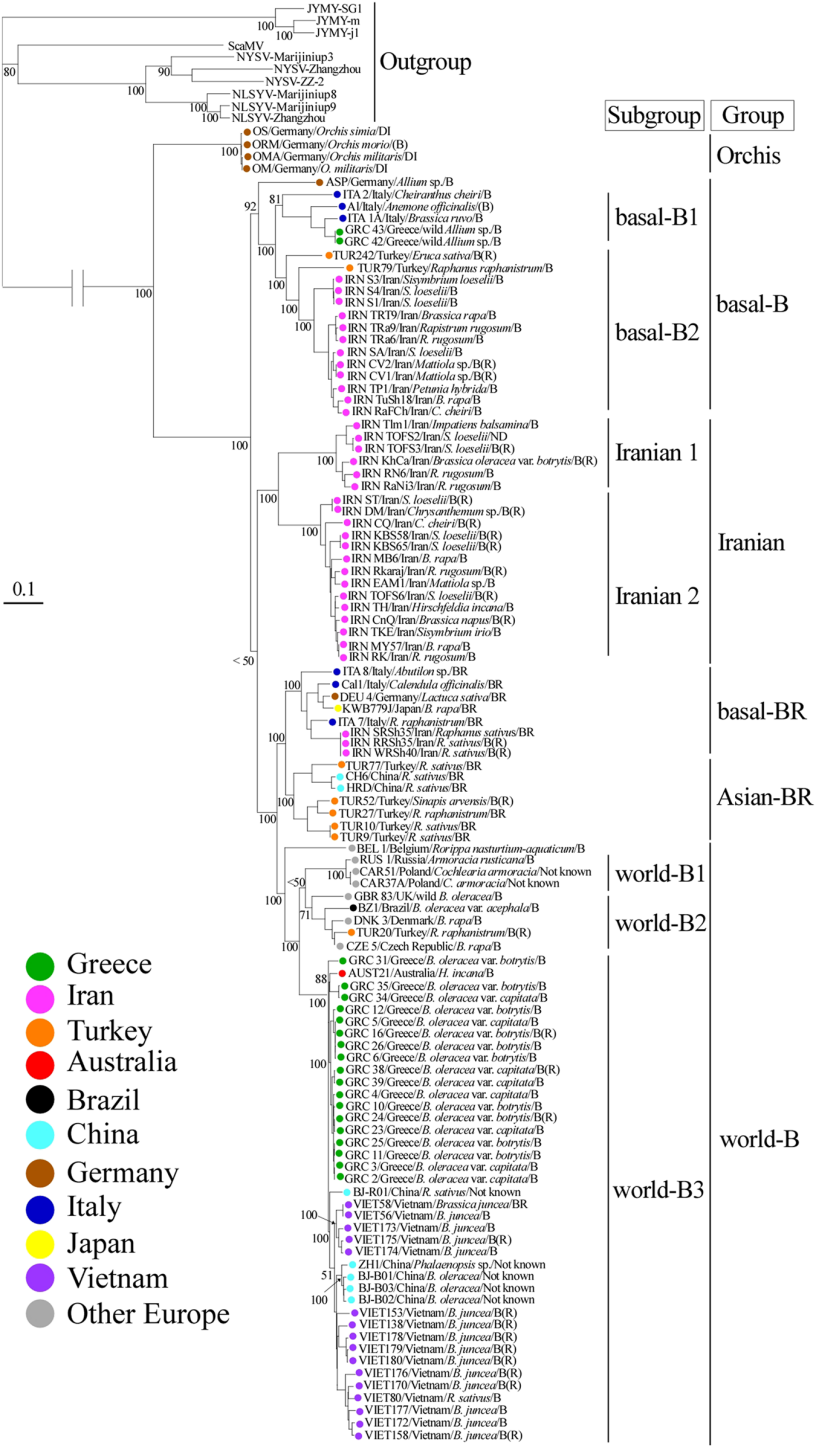
Maximum-likelihood tree inferred from the major open reading fame sequences of turnip mosaic virus. Only non-recombinant sequences were used. Numbers at each node indicate bootstrap percentages based on 1000 pseudoreplicates. The scale bar indicates 0.1 substitutions per site. The genomic sequence of the isolates of narcissus late season yellows virus (NLSYV), narcissus yellow stripe virus (NYSV), Japanese yam mosaic virus (JYMV), and scallion mosaic virus (ScaMV) were used as outgroup taxa. Details of the isolates are given in Supplementary Table S1.

We inferred a maximum-likelihood phylogenetic tree using the polyprotein-encoding (major ORF) sequences of the non-recombinants (Fig. 3) together with isolates represented by the three regions that contained no recombination cross-over points in any sequence: HC-Pro* (nt 1460–2494, numbers corresponding to the positions in original UK 1 genome; partial HC-Pro), P3* (nt 2591–3463; partial P3) and NIb* (nt 7208–8068; partial NIb) (see Yasaka *et al*.^11^). Trees were estimated using 420, 410 and 423 non-recombinant sequences, respectively (data not shown). These partitioned most of the sequences into the same five major genetic groups that were reported previously^10, 11^, Orchis, basal-B, basal-BR, Asian-BR, and world-B groups, and a new Iranian group. The basal-B group further split into basal-B1 and B2 subgroups and the world-B group split into the world-B1, B2 and B3 subgroups, as found in an earlier study^11^. In the present study, many non-recombinant sequences in TuMV population were found for the first time.

### Time of TuMV emergence

We found that there was little saturation across the TuMV protein sequences in our data sets, based on our analyses of the aligned ORF sequences using the Iss statistic in DAMBE^27^. The estimates of Iss were significantly lower than Iss.c for all data sets, and were 4–5 times lower for major ORF, HC-Pro*, P3*, and NIb* sequences.

Using a Bayesian phylogenetic approach, we estimated the evolutionary rates and timescales for the complete major ORF, HC-Pro*, P3* and NIb* regions. We found 106 non-recombinants in this study, so we used these to provide ORF sequences for analysis. The HC-Pro*, P3* and NIb* regions were shorter than major ORF sequences, but many more sequences were available (329, 369 and 351, respectively).

Based on a comparison of marginal likelihoods, the constant-size demographic model was the best supported for all four proteins (Table 1). An uncorrelated exponential relaxed-clock model^28^ provided a better fit than the strict-clock model, indicating the presence of rate variation among lineages. All data sets passed date-randomization tests for temporal structure^8–10, 29, 30^.

**Table 1:** Estimates of nucleotide substitution rate and time to the most recent common ancestor for turnip mosaic virus.

Parameter	Protein-coding region			
	Major ORF	HC-Pro*	P3*	NIb*
a. All sites				
Sequence length (nt)	9432	927	873	855
Sampling date range	1968–2012	1968–2014	1968–2014	1968–2014
TMRCA^b^ (years)				
All isolates	1201 (468–2150)^c^ (n = 106)	951 (326–1291) (n = 329)	758 (274–1548) (n = 369)	1080 (318–1605) (n = 351)
basal-B group	321 (173–520) (n = 21)	163 (79–308) (n = 102)	174 (81–345) (n = 105)	190 (86–361) (n = 81)
basal-B1 subgroup	252 (160–356) (n = 5)	ND^d^ (n = 12)	ND (n = 12)	ND (n = 12)
basal-B2 subgroup	235 (154–345) (n = 15)	114 (59–268) (n = 93)	129 (75–299) (n = 93)	141 (75–300) (n = 68)
Iranian group	178 (95–233) (n = 20)	162 (69–305) (n = 35)	142 (62–295) (n = 35)	173 (81–314) (n = 35)
Iranian 1 subgroup	102 (49–175) (n = 6)	90 (33–140) (n = 7)	87 (37–125) (n = 7)	89 (35–146) (n = 7)
Iranian 2 subgroup	140 (75–207) (n = 14)	101 (45–158) (n = 28)	111 (42–183) (n = 98)	124 (57–191) (n = 28)
basal-BR group	228 (141–344) (n = 8)	167 (46–267) (n = 15)	ND (n = 17)	ND (n = 19)
Asian BR group	234 (145–337) (n = 7)	198 (57–279) (n = 45)	125 (47–261) (n = 69)	129 (61–244) (n = 59)
world-B group	321 (204–479) (n = 46)	181 (59–293) (n = 130)	135 (50–192) (n = 139)	153 (80–279) (n = 153)
world-B1 subgroup	ND (n = 3)	ND (n = 6)	ND (n = 6)	ND (n = 5)
world-B2 subgroup	ND (n = 5)	154 (40–222) (n = 39)	86 (42–176) (n = 21)	107 (62–192) (n = 27)
world-B3 subgroup	152 (70–205) (n = 37)	121 (32–189) (n = 83)	72 (31–119) (n = 110)	87 (36–127) (n = 120)
Substitution rate (nt/site/year)				
All isolates	8.89 × 10^−4^ (6.87 × 10^−4^–1.30 × 10^−3^)	1.41 × 10^−3^ (1.09 × 10^−3^–1.78 × 10^−3^)	1.46 × 10^−3^ (1.25 × 10^−3^–1.87 × 10^−3^)	1.37 × 10^−3^ (1.04 × 10^−3^–1.73 × 10^−3^)
basal-B group	8.14 × 10^−4^ (6.70 × 10^−4^–1.25 × 10^−3^)	1.27 × 10^−3^ (9.02 × 10^−4^–2.13 × 10^−3^)	1.63 × 10^−3^ (8.29 × 10^−4^–3.24 × 10^−3^)	9.26 × 10^−4^ (7.91 × 10^−4^–2.51 × 10^−3^)
basal-B1 subgroup	9.23 × 10^−4^ (7.90 × 10^−4^–1.58 × 10^−3^)	ND	ND	ND
basal-B2 subgroup	7.53 × 10^−4^ (5.20 × 10^−4^–9.62 × 10^−3^)	1.58 × 10^−3^ (8.56 × 10^−4^–3.41 × 10^−3^)	1.39 × 10^−3^ (9.11 × 10^−4^–2.46 × 10^−3^)	1.25 × 10^−3^ (9.01 × 10^−4^–2.03 × 10^−3^)
Iranian group	8.20 × 10^−4^ (6.84 × 10^−4^–1.53 × 10^−3^)	1.19 × 10^−3^ (6.23 × 10^−4^–2.34 × 10^−3^)	1.28 × 10^−3^ (7.22 × 10^−4^–3.01 × 10^−3^)	1.38 × 10^−3^ (8.01 × 10^−4^–3.12 × 10^−3^)
Iranian 1 subgroup	9.31 × 10^−4^ (7.14 × 10^−4^–1.41 × 10^−3^)	1.32 × 10^−3^ (7.58 × 10^−4^–2.22 × 10^−3^)	1.44 × 10^−3^ (1.08 × 10^−3^–2.56 × 10^−3^)	1.26 × 10^−3^ (9.08 × 10^−4^–2.58 × 10^−3^)
Iranian 2 subgroup	9.32 × 10^−4^ (7.11 × 10^−4^–1.48 × 10^−3^)	1.22 × 10^−3^ (7.31 × 10^−4^–2.51 × 10^−3^)	1.15 × 10^−3^ (8.97 × 10^−4^–2.67 × 10^−3^)	1.08 × 10^−3^ (9.22 × 10^−4^–2.18 × 10^−3^)
basal-BR group	1.65 × 10^−3^ (8.98 × 10^−4^–2.03 × 10^−3^)	2.08 × 10^−3^ (8.14 × 10^−4^–3.54 × 10^−3^)	ND	ND
Asian BR group	1.33 × 10^−3^ (9.04 × 10^−4^–1.78 × 10^−3^)	1.16 × 10^−3^ (5.99 × 10^−4^–1.98 × 10^−3^)	1.55 × 10^−3^ (8.62 × 10^−4^–2.94 × 10^−3^)	1.29 × 10^−3^ (8.07 × 10^−4^–3.05 × 10^−3^)
world-B group	1.10 × 10^−3^ (7.99 × 10^−4^–1.77 × 10^−3^)	1.68 × 10^−3^ (7.02 × 10^−4^–3.25 × 10^−3^)	1.02 × 10^−3^ (5.96 × 10^−4^–2.81 × 10^−3^)	9.22 × 10^−4^ (4.93 × 10^−4^–2.15 × 10^−3^)
world-B1 subgroup	ND	ND	ND	ND
world-B2 subgroup	ND	1.35 × 10^−3^ (5.08 × 10^−4^–2.10 × 10^−3^)	9.16 × 10^−4^ (3.87 × 10^−4^–1.84 × 10^−3^)	7.45 × 10^−4^ (3.27 × 10^−4^–1.44 × 10^−3^)
world-B3 subgroup	9.20 × 10^−4^ (5.24 × 10^−4^–1.69 × 10^−3^)	2.02 × 10^−3^ (9.77 × 10^−4^–3.05 × 10^−3^)	2.51 × 10^−3^ (1.25 × 10^−3^–2.97 × 10^−3^)	1.79 × 10^−3^ (1.02 × 10^−3^–3.69 × 10^−3^)
dN/dS^e^	0.062	0.022	0.126	0.026
No. of variable sites^f^	4653/9432 (49%)	421/927 (45%)	556/873 (64%)	428/855 (50%)
**b. Synonymous sites**				
Sequence length (nt)	6078	525	300	519
Sampling date range	1968–2012	1968–2014	1968–2014	1968–2014
TMRCA (years)				
All isolates	1570 (521–3430)	1059 (549–1401)	1134 (646–1867)	1178 (635–1791)
basal-B group	389 (200–676)	276 (181–472)	254 (136–421)	271 (140–463)
basal-B1 subgroup	ND	ND	ND	ND
basal-B2 subgroup	321 (210–434)	252 (169–439)	220 (116–379)	238 (139–442)
Iranian group	247 (155–338)	152 (69–229)	172 (63–267)	139 (57–206)
Iranian 1 subgroup	130 (69–202)	79 (36–178)	107 (55–169)	105 (47–151)
Iranian 2 subgroup	144 (83–217)	122 (56–202)	129 (54–213)	114 (48–205)
basal-BR group	269 (184–363)	204 (110–374)	ND	ND
Asian BR group	ND	276 (174–445)	222 (129–403)	199 (100–324)
world-B group	373 (235–507)	232 (119–392)	205 (93–381)	220 (102–389)
world-B1 subgroup	ND	ND	ND	ND
world-B2 subgroup	ND	190 (88–324)	158 (75–252)	181 (81–297)
world-B3 subgroup	195 (106–297)	138 (79–241)	124 (75–199)	111 (63–174)
Substitution rate (nt/site/year)				
All isolates	8.22 × 10^−4^ (6.25 × 10^−4^–1.56 × 10^−3^)	1.45 × 10^−3^ (1.13 × 10^−3^–1.81 × 10^−3^)	1.65 × 10^−3^ (1.29 × 10^−3^–2.01 × 10^−3^)	1.36 × 10^−3^ (9.91 × 10^−4^–1.72 × 10^−3^)
basal-B group	8.55 × 10^−4^ (4.23 × 10^−4^–3.26 × 10^−3^)	1.23 × 10^−3^ (6.14 × 10^−4^–3.92 × 10^−3^)	1.71 × 10^−3^ (5.29 × 10^−4^–3.14 × 10^−3^)	1.62 × 10^−3^ (6.23 × 10^−4^–2.94 × 10^−3^)
basal-B1 subgroup	ND	ND	ND	ND
basal-B2 subgroup	7.21 × 10^−4^ (3.93 × 10^−4^–1.31 × 10^−3^)	9.13 × 10^−4^ (2.60 × 10^−4^–1.75 × 10^−3^)	1.12 × 10^−3^ (3.12 × 10^−4^–2.22 × 10^−3^)	1.23 × 10^−3^ (2.64 × 10^−4^–2.15 × 10^−3^)
Iranian group	1.02 × 10^−3^ (6.94 × 10^−4^–2.14 × 10^−3^)	1.21 × 10^−3^ (6.21 × 10^−4^–4.11 × 10^−3^)	2.35 × 10^−3^ (4.65 × 10^−4^–4.25 × 10^−3^)	1.79 × 10^−3^ (3.11 × 10^−4^–4.01 × 10^−3^)
b. Synonymous sites				
Iranian 1 subgroup	9.24 × 10^−4^ (5.22 × 10^−4^–2.23 × 10^−3^)	1.28 × 10^−3^ (4.02 × 10^−4^–3.21 × 10^−3^)	2.08 × 10^−3^ (3.55 × 10^−4^–3.58 × 10^−3^)	1.62 × 10^−3^ (3.99 × 10^−4^–5.07 × 10^−3^)
Iranian 2 subgroup	8.10 × 10^−4^ (4.31 × 10^−4^–1.67 × 10^−3^)	1.41 × 10^−3^ (2.47 × 10^−4^–3.31 × 10^−3^)	2.35 × 10^−3^ (2.47 × 10^−4^–3.59 × 10^−3^)	1.45 × 10^−3^ (6.21 × 10^−4^–3.61 × 10^−3^)
basal-BR group	1.54 × 10^−3^ (7.13 × 10^−4^–3.24 × 10^−3^)	2.66 × 10^−3^ (1.10 × 10^−3^–3.48 × 10^−3^)	ND	ND
Asian BR group	ND	1.38 × 10^−3^ (1.92 × 10^−4^–3.01 × 10^−3^)	1.89 × 10^−3^ (2.76 × 10^−4^–3.87 × 10^−3^)	1.58 × 10^−3^ (3.05 × 10^−4^–4.02 × 10^−3^)
world-B group	1.25 × 10^−3^ (9.01 × 10^−4^–2.25 × 10^−3^)	1.65 × 10^−3^ (9.75 × 10^−4^–3.21 × 10^−3^)	9.15 × 10^−4^ (6.25 × 10^−4^–1.49 × 10^−3^)	1.05 × 10^−3^ (8.24 × 10^−4^–2.11 × 10^−3^)
world-B1 subgroup	ND	ND	ND	ND
world-B2 subgroup	ND	9.89 × 10^−4^ (3.74 × 10^−4^–1.48 × 10^−3^)	8.56 × 10^−4^ (3.02 × 10^−4^–1.35 × 10^−3^)	9.03 × 10^−4^ (2.74 × 10^−4^–1.57 × 10^−3^)
world-B3 subgroup	8.16 × 10^−4^ (4.24 × 10^−4^–1.53 × 10^−3^)	2.47 × 10^−3^ (1.62 × 10^−3^–3.08 × 10^−3^)	3.41 × 10^−3^ (1.12 × 10^−3^–3.77 × 10^−3^)	2.59 × 10^−3^ (1.12 × 10^−3^–3.58 × 10^−3^)
No. of variable sites	2095/6078 (34%)	195/525 (37%)	110/300 (37%)	191/519 (37%)

^a^Complete major ORF (open reading frame) (polyprotein), HC-Pro* (partial helper-component proteinase), P3* (partial protein 3) and NIb* (partial nuclear inclusion b) regions.

^b^Time to the most recent common ancestor (years before 2012).

^c^95% credibility intervals in parentheses.

^d^Not determined. The data set did not pass the date-randomization test.

^e^Non-synonymous (dN) and synonymous (dS) substitution (dN/dS) ratios were calculated for four proteins.

^f^The number of variable sites/total sites.

The time to the most recent common ancestor (TMRCA) for each of the four protein-coding regions (major ORF, HC-Pro*, P3* and NIb*) was estimated using all sites and found to be 998 years on average (Table 1a). Mean estimates from individual protein-coding regions ranged from 1201 years [95% credibility intervals (CIs) 468–2150] years for the major ORF to 758 (95% CI 274–1548) years for P3*. The estimates had overlapping 95% CIs for rates and TMRCAs. However, the 1.6-fold range of estimated mean TMRCAs compromised their ability to distinguish among historical events that might have influenced TuMV evolution.

**Figure 4:**
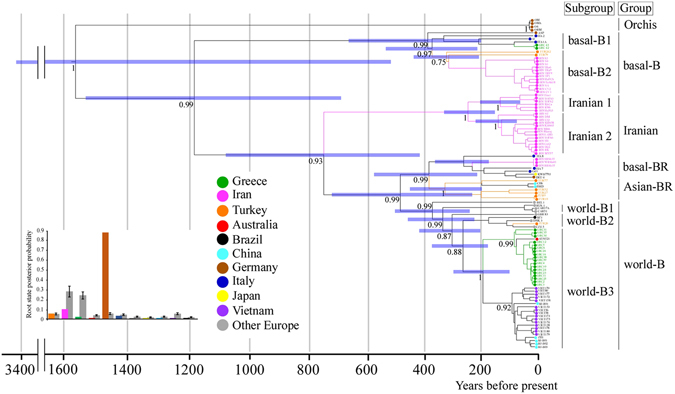
Bayesian maximum-clade-credibility chronogram inferred from the polyprotein-coding region of turnip mosaic virus genomes. The tree was estimated from the major open reading frame (ORF) sequences of 106 non-recombinant isolates. Detail of the region is given in the Methods. Horizontal blue bars represent the 95% credibility intervals of estimates of node ages. The bar graph shows the root state posterior probabilities for each location. Grey bars show the probabilities obtained with 10 randomizations of the tip locations. Year before present; 2012.

We also checked whether using only the synonymous sites in the sequences decreased the variability of the results (Table 1b). The effect of limiting the analysis to the synonymous sites is to minimize the influence of purifying selection, which can otherwise lead to an underestimation of TMRCAs when sampling dates are used for calibration^31, 32^. The synonymous sites from these four proteins passed the date-randomization test. Bayesian maximum-clade-credibility (MCC) chronograms of major ORF, HC-Pro*, P3* and NIb* were inferred from synonymous sites (Fig. 4 and Supplementary Fig. S2). The TMRCA of the major ORF region was 1570 (95% CI 521–3430) years, and those of three shorter protein-coding regions were 1059–1178 years (95% CI 549–1867 years). The ranges of the estimates were similar to those from the whole sequences (Table 1).

### Geographical spread of TuMV

**Figure 5:**
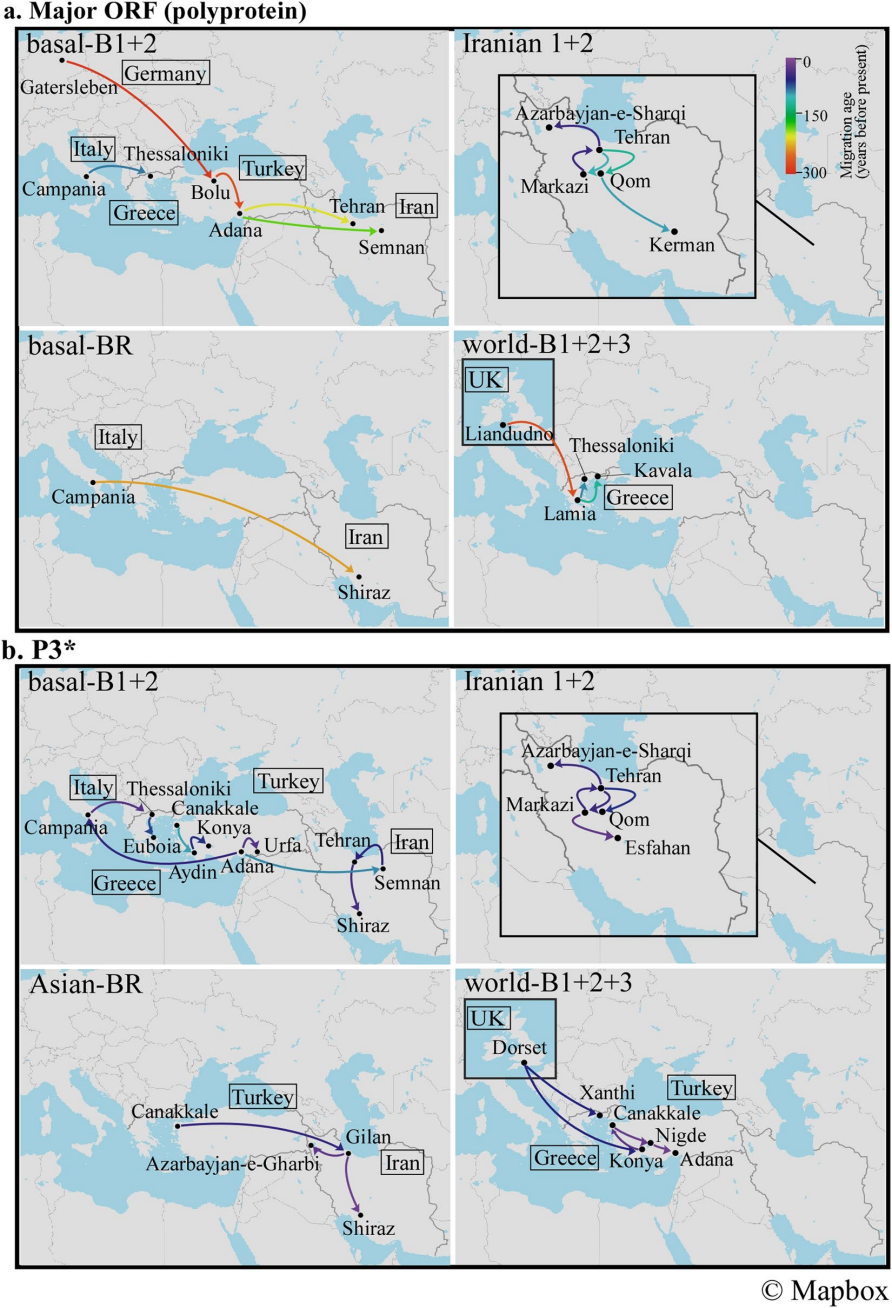
Plausible historical dissemination pathways of turnip mosaic virus inferred using the major open reading frame (ORF) and partial protein 3 (P3*) sequences using non-recombinant sequences. Details of the regions of (a) major ORF and (b) P3* are given in the Methods. Dissemination routes are only shown for the Middle East, and only when supported by a Bayes factor >10. The dissemination pathways for basal-B1 +2, Iranian 1 + 2, basal-BR, Asian-BR and world-B1 +2 + 3 group (subgroup) isolates are shown (https://www.mapbox.com/about/maps/).

The likely routes of TuMV dissemination in/into Turkey, Greece and Iran were assessed using a Bayesian phylogeographical analysis^33^, based on the non-recombinant sequences of the major ORF, HC-Pro*, P3* and NIb* regions (Figs 4 and 5 and Supplementary Figs S2 and S3; Supplementary Table S3). The major ORF data sets contained no recombination cross-over points from non-recombinant isolates, whereas the HC-Pro*, P3* and NIb* data sets also contained no recombination cross-over point sequences but from both non- and recombinant isolates. The partial-genome data sets contained at least three times as many sequences as the ORF data sets, but the optimal trade-off between sequence length and number remains unclear. Additionally, the major ORF data set yields evidence of the dissemination of non-recombinants, whereas the HC-Pro*, P3* and NIb* data sets provides evidence about the dissemination of both non-recombinants and partial-genome sequences in recombinants.

**Figure 6:**
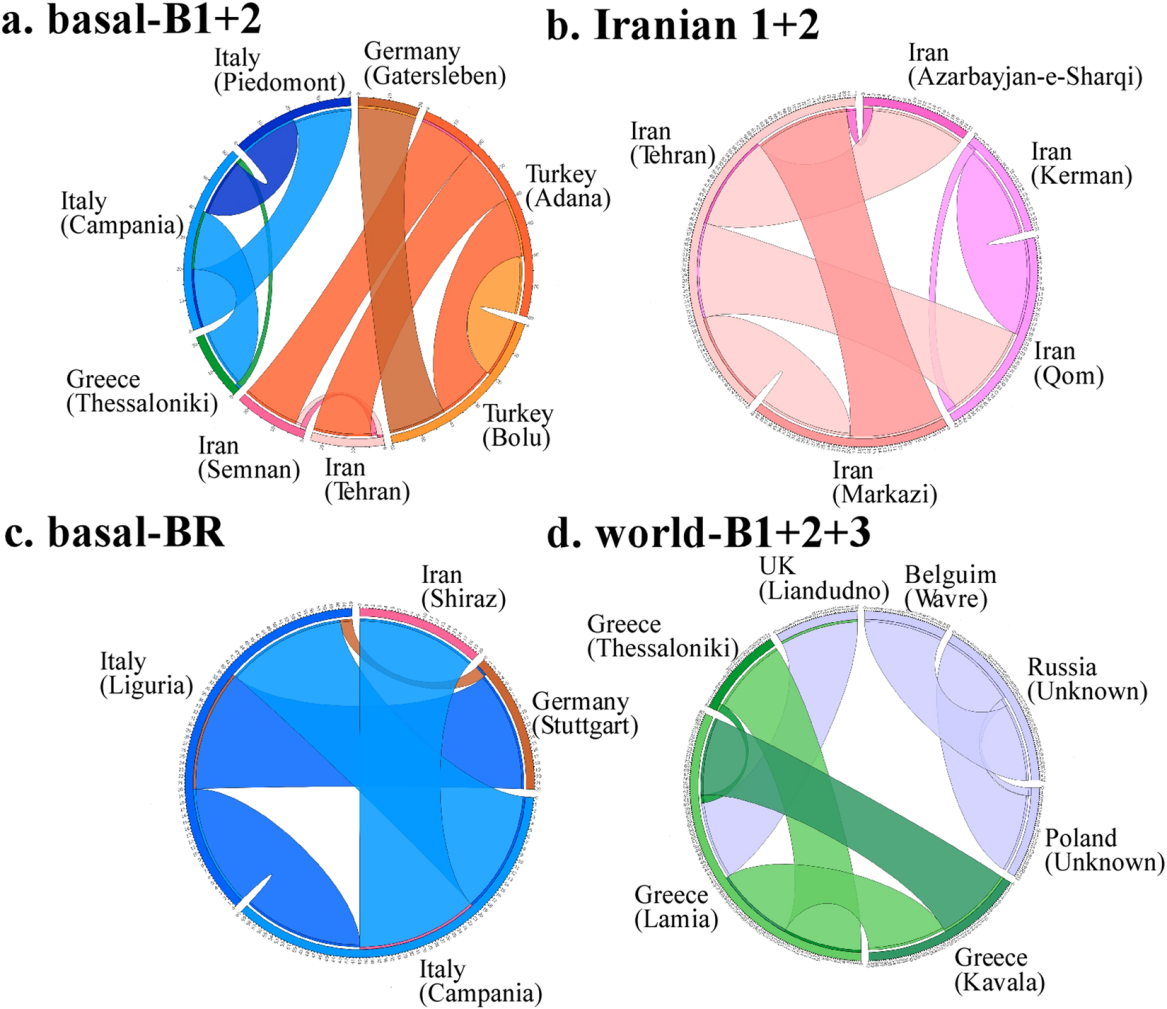
Predicted dissemination events between Middle Eastern countries and other countries using the major open reading frame sequences of turnip mosaic virus. Lines indicate dissemination events between and within pairs of countries and cities, with colors indicating the source state. The colors of the inner and outer circles show the source and sink cities. Only the dissemination pathways for (a) basal-B1 +2, (b) Iranian 1 + 2, (c) basal-BR and (d) world-B1 +2 + 3 group (subgroup) isolates are shown. Narrow links indicate dissemination events that are not statistically significant. Bold links indicate dissemination events with *P* < 0.05.

We investigated the routes of spread for each TuMV phylogenetic group or subgroup. In the partial-genome data sets, TuMV seems to have circulated not only within each country (Greece, Iran and Turkey), but also between Turkey and Greece, between Turkey and Iran, and between Turkey and Italy. The last of these is also supported by the major ORF data set. Spread from Germany to Turkey was found in the major ORF data, but not in HC-Pro*, P3* or NIb*; the isolates from orchids and *Allium* sp. plants were found in Germany and spread earlier than elsewhere (298 years ago, 95% CI 121–429). By contrast, the world-B group isolates spread in all three countries and the UK was also involved. The results were confirmed in the maximum-likelihood and Bayesian trees of the major ORF, HC-Pro*, P3* and NIb* regions (Figs 3 and 4 and Supplementary Fig. S2). In addition, most of these disseminations were supported by the results of the MigraPhyla program^34^ using the same data set of major ORFs including only non-recombinants (Fig. 6). The spread between Turkey and Greece and between Turkey and Iran were seen, and between Europe and these countries. The results of all analyses supported the conclusion that TuMV had entered the Middle East from the west and had progressively spread eastwards.

### Timescale of TuMV groups and recombination

In both the analyses of all sites and synonymous sites in the major ORF coding region, the basal-B group seemed to be the oldest lineage of TuMV (excluding the Orchis group). For instance, the TMRCA of the basal-B group by synonymous site analysis in the ORF coding regions was 389 (95% CI 200–676) years. The basal-B group was placed as the sister lineage to other brassica-infecting phylogenetic groups^10^, as seen in the maximum-likelihood and Bayesian ORF trees (Figs 3 and 4). We also estimated the diversity and extent of negative selection on the sequences in each phylogenetic group. The basal-B group has the greatest diversity, although the strength of selection was similar across all phylogenetic groups.

We estimated the ages of recombination events using the method described by Visser *et al*.^12^ and Yasaka *et al*.^11^. Recombinant sequences were split into their separate regions and realigned using gaps. For example, a recombinant with two ‘parents’ was split into two regions and the empty sites were filled with gaps. In this way, a recombinant sequence becomes two non-recombinant sequences, each with missing data. The oldest recombination event that was detected occurred 188 (95% CI 153–222) years ago in Turkey and produced an intralineage recombinant of basal-B2 parents (Table 2). The six oldest recombination events were all intralineage recombinants of basal-B2 subgroup parents in Greece and Turkey. The three oldest recombination cross-over points were located at nt 6222 in VPg, nt 7120 in NIa-Pro and nt 8963 in CP coding regions.

**Table 2:** Estimates of the timing of recombination events of turnip mosaic virus in Greece, Iran and Turkey.

Recombination age (YBP)	Stem age (YBP)	Crown age (YBP)	Recombination site^a^	Recombinant type^b^	Parent (5′ × 3′)	Country
188 (153–222)^c^	195 (168–222)^d^	184 (153–215)	nt 6222	Intra	basal-B2 × basal-B2	Turkey
186 (123–248)	201 (153–248)	165 (123–206)	nt 7120	Intra	basal-B2 × basal-B2	Greece
183 (149–216)	191 (165–216)	177 (149–205)	nt 7120	Intra	basal-B2 × basal-B2	Turkey
180 (129–228)	208 (136–228)	162 (129–195)	nt 8963	Intra	basal-B2 × basal-B2	Greece
156 (132–179)	208 (136–179)	147 (132–161)	nt 1202	Intra	basal-B2 × basal-B2	Turkey
148 (123–172)	153 (133–172)	141 (123–158)	nt 8112	Intra	basal-B2 × basal-B2	Turkey
141 (119–162)	143 (124–162)	134 (119–149)	nt 706	Inter	Asian-BR × basal-B2	Turkey
135 (108–161)	137 (113–161)	129 (108–149)	nt 2523	Intra	basal-B2 × basal-B2	Greece
131 (109–152)	138 (123–152)	120 (109–131)	nt 706	Inter	Asian-BR × basal-B2	Greece
115 (89–141)	129 (116–141)	103 (89–116)	nt 1455	Intra	Iranian 2 × Iranian 2	Iran

^a^The age of recombination sites in Greece, Iran and Turkey were estimated with reference to the results of our Bayesian phylogenetic analyses. Only the oldest ten recombination sites are listed. The common recombination sites in these countries were estimated from the tree including all isolates (data not shown). Nucleotide positions show locations of individual genes numbered as in the original UK 1 genome^57^.

^b^Inter-, interlineage recombination site; intra, intralineage recombination site.

^c^The youngest and oldest ages are shown for recombination age. The youngest and oldest ages were estimated from the stem and crown ages, respectively. Estimates are given in YBP (years before present; 2012).

^d^95% credibility intervals are shown in parentheses for stem and crown ages.

## Discussion

We have reported the most detailed evolutionary study of isolates from the centre of emergence of a plant virus, based on a global sample of more than 400 whole-genome sequences of TuMV (approximately 10,000 nt). We previously reported genetic analyses of the TuMV populations in Europe, East Asia and Oceania. However, the centre of emergence is thought to be in the populations of the Middle East, which remain largely uncharacterized. Our earlier studies^10, 19^ reported that approximately 75% of isolates from TuMV populations are recombinants. Therefore, to resolve the evolutionary history of this virus, we must analyse non-recombinants, especially from its centre of emergence. The many non-recombinants that we have identified in this study have allowed us to resolve the possible dissemination routes of this virus, along with the genetic changes that occurred as it adapted to new hosts and moved to other parts of the world.

In this study, we found a sixth phylogenetic group of TuMVs, the Iranian group. This adds to the previously described Orchis, basal-B, basal-BR, Asian-BR and world-B groups. The Orchis group consists of isolates from Europe (Germany) and is probably the original lineage of TuMV. The basal-B group is probably the sister lineage to the remaining groups and splits into two subgroups: the basal-B1 subgroup, which consists of isolates from Europe (Italy and Greece); and the basal-B2 subgroup, which consists of Middle East isolates (Turkey and Iran). Although each subgroup is geographically restricted, the basal-B1 subgroup seems to be the oldest modern subgroup, because many basal-B2 group isolates were recombinants whereas basal-B1 isolates were not (Fig. 2). The isolate ASP from *Allium* sp. is resolved as the sister lineage to all basal-B isolates. Further sampling of TuMV lineages, particularly from monocotyledonous plants, is needed to determine the history of adaptation that led to the divergence into the Orchis lineage and the brassica-infecting lineage.

We were unable to find TuMV in wild orchids in Greece, Turkey and Iran in the present study. Thus, ORF trees inferred using non-recombinant sequences still indicated that TuMV infecting brassicas might have originated from ancestral populations in wild orchids, *Orchis militalis*, *O. morio* and *O. simia* ^10^. Although the wild orchids were collected in Northern Germany, it is unclear whether the wild orchids were infected with TuMV-OMs^10^ in Germany or in other European countries. This is because various species of wild orchids are widely distributed in European countries and, as they are bulbs, they are often transported by plant collectors, and both the Orchis-infecting and *Allium*-infecting isolates came from an orchid collection in Gatersleben, Germany (Supplementary Table S1). This country is probably the source of TuMV basal-B group and might be the site of origin of TuMV. The virus might then have spread to Italy and Greece, and infected wild brassicas, and from there to Turkey and Iran. Denser sampling of the TuMV lineages in these groups will shed further light on these questions.

At or after the emergence of basal-B, TuMV spread to Iran and split into two subgroups. Because TuMV has not yet been collected from the neighbouring countries of Afghanistan, Iraq and Pakistan, we suspect that the Iranian groups are unique.

The BEL 1 isolate collected from *Rorippa nasturtium-aquaticum* (watercress) was placed as the sister lineage to all other world-B isolates. *Rorippa* is perennial and thought to have originated in Europe and Central Asia. However, the trees do not tell us the origin of world-B group, hence more isolates of the group need to be collected to answer this question.

Non-recombinant isolates from the Asian-BR group that infect *R. sativus* (radish) were previously found in China^19^. In this study, however, some Asian-BR non-recombinants were found in Turkey. Hence, the Asian-BR group might have originated in Turkey (Fig. 3), which is considered also to be one of origins of wild radish (*R. raphanistrum*). In fact, we saw many wild radish plants in the fields along the shore of the Aegean Sea during our collecting trips (K. Ohshima and S. Korkmaz, personal observation). However, our Bayesian phylogeographic analyses only found that the Asian-BR subgroup spread in Iran and from Turkey to Iran (Supplementary Table S3), and thence to southern Asia, where radish is one of the major crops and important for Asian cuisine.

Another group of isolates that infect radish belong to the basal-BR group. This modern group of isolates possibly originated in Italy, given the phylogenetic distribution of Italian lineages within the group. Other isolates have been found in Germany, Iran and Japan. No non-recombinants of the basal-BR group have been found in China, so we are unsure whether the dissemination route of this group to East Asia is the same as that of the Asian-BR group; more samples of TuMV from Central Asian countries are needed to answer this question. The basal-BR and Asian-BR populations might have spread to the east in plant material carried along the Northern or Southern Silk roads, an ancient network of trade routes between the Mediterranean and East Asia. However, our analyses indicate that different TuMV populations seem to have spread individually to different parts of the world.

Our estimation of the evolutionary and phylogeographic timescale was based on complete sequences as well as the synonymous sites. This approach was also previously used for estimating TMRCAs for CMV^8^. There are small differences between our two estimates of the evolutionary timescale. The mean TMRCA estimates from the synonymous sites were less variable than those from the complete sequences (Table 1). The longer sequences of the major ORF might give us a more reliable estimate of the TMRCA. However, the shorter sequences of HC-Pro*, P3* and NIb* yielded consistent estimates of the TMRCAs, and these three regions had three times as many isolates as the major ORF.

If the ancestors of the present TuMV populations depended on agricultural practices for their maintenance and spread, such as the collection and transport of TuMV-infected seed, then the estimated TMRCAs set limits on when brassicas were adopted as agricultural crops. The emergence of the brassica-infecting group corresponds well with the periods of territorial expansion of the Ottoman Empire in Greece, Turkey and Iran, and the spread of agriculture to the world.

## Methods

### Virus isolates and host tests

The brassica crop-producing areas of Greece, Iran and Turkey were surveyed during the growing seasons of 1993–2012. All of the collected plant leaves were tested by double-antibody sandwich enzyme-linked immunosorbent assay (DAS-ELISA)^35^ using the antiserum to TuMV^9^. The virus isolates were found in fields as well as in home gardens. In Turkey, wild *Raphanus* plants are common, and diseased plants were relatively easy to find. Thus, 27 Turkish isolates were collected from wild plants (*R. raphanistrum*) and crops (*R. sativus*), 25 isolates from brassicas, and six from other species of Brassicaceae. The Greek samples included 27 from *Brassica* spp., 15 from other *Brassicaceae* plants and four from *Allium* spp. In Iran, we were able to collect many brassica plants throughout the country, but not from from the border regions because of the armed conflict occurring there. Details of the TuMV isolates, their place of origin, original host plant, year of collection, host-infecting type, accession numbers, and references are shown in Supplementary Table S1.

All of the isolates were sap-inoculated to *Chenopodium quinoa* plants using 0.01 M potassium phosphate buffer (PPB) (pH 7.0) and serially cloned through single lesions at least three times using chlorotic local lesions that appeared approximately 10 days after inoculation. The biological cloning step is important because TuMV isolates were often co-infected with CMV and/or CaMV, and some plants contained a mixture of different TuMV isolates. Hence, there is a possibility that artificial recombination events will be detected in the sequence data from uncloned isolates. Biologically purified TuMV isolates were propagated in *Nicotiana benthamiana* and *B*. *rapa* cv. Hakatasuwari (turnip) plants. Plants infected systemically with each of the TuMV isolates were homogenized in 0.01 M PPB (pH 7.0), and the isolates were mechanically inoculated to young brassica plants, as described by Nguyen *et al*.^10^. Inoculated plants were kept at 25 °C for at least four weeks in a glasshouse at Saga University.

### Sequencing and alignment

We determined the full genomic sequences of 179 TuMV isolates collected in Greece, Iran and Turkey. The viral RNAs were extracted from TuMV-infected *N. benthamiana* or turnip leaves using Isogen (Nippon Gene, Japan). The RNAs were reverse transcribed by PrimeScript Moloney murine leukemia virus reverse transcriptase (Takara Bio, Japan) and amplified using high-fidelity Platinum™ Pfx DNA polymerase (Invitrogen, USA). The products obtained by reverse transcription and polymerase chain reaction (RT-PCR) were separated by electrophoresis in agarose gels and purified using the QIAquick Gel Extraction Kit (Qiagen K. K., Japan).

Sequences from each isolate were determined using three or four overlapping independent RT-PCR products to cover the complete genome. To ensure that they were from the same genome and were not from different components of a genome mixture, the sequences of the RT-PCR products of adjacent regions of the genome overlapped by 200–350 nt. Each RT-PCR product was sequenced by primer walking in both directions using a BigDye Terminator v3.1 Cycle Sequencing Ready Reaction kit (Life Technologies, USA) and an Applied Biosystems 310 and 3130 Genetic Analyzer. Sequence data were assembled using BioEdit v5.0.9^36^.

We assembled a data set of 417 genome sequences (Supplementary Table S1), comprising the 179 sequences determined in this study and 238 published sequences from online databases (collected in September 2015). The genomic sequences of the isolates of narcissus late season yellows virus (NLSYV; accession numbers JQ326210, JX156421 and NC_023628), narcissus yellow stripe virus (NYSV; JQ395042, JQ911732 and NC_011541), Japanese yam mosaic virus (JYMV; AB016500, KJ789140 and NC_000947) and scallion mosaic virus (ScaMV; NC_003399) were used as outgroup taxa because those viruses are members of TuMV phylogenetic group.

The nucleotide sequences of the polyprotein-encoding regions were aligned using TRANSALIGN (kindly supplied by Georg Weiller) and their encoded amino acid sequences aligned using CLUSTAL_X2^37^. The aligned nucleotides were then reassembled to form whole-genome sequences by adding the aligned 5′ and 3′ NCR regions of RNA. This produced sequences of 9051 nt that excluded the 35 nucleotides that were used as primers for RT-PCR amplification.

### Recombination analyses

Putative recombination breakpoints in all sequences were identified using RDP^38^, GENECONV^39^, BOOTSCAN^40^, MAXCHI^41^, CHIMAERA^42^ and SISCAN^43^ programs, implemented in the RDP4 package^44^, and also the original SISCAN v2^43^ program. Each of the identified sites was examined individually, and a phylogenetic approach was used to verify the parent/donor assignments made using the RDP4 package^44^. These analyses were done using default settings for the different detection programs and a Bonferroni-corrected *P*-value cut-off of 0.01.

We tested for recombination in our data set of 417 genome sequences. Having examined all sites with an associated *P*-value of < 10^−6^ (i.e., the most likely recombination sites), we retained the intralineage recombinants (parents from the same major group lineage) and removed the interlineage recombinants (i.e., those with parents from different major lineages). The identified recombination sites were treated as missing data in subsequent analyses. All isolates that had been identified as likely recombinants by the programs in RDP4, supported by three different methods with an associated *P*-value of > 10^−6^, were rechecked using the original SISCAN program. We checked 50 nt slices of all sequences for evidence of recombination using these programs. These analyses also determined which non-recombinant sequences had regions that were closest to those of the recombinant sequences and hence indicated the lineages that were likely to have provided those regions of the recombinant genomes. For convenience, we called these the ‘parental isolates’ of the recombinants. Finally, TuMV sequences were also aligned without outgroup sequences, producing sequences of 9693 nt for full genome RNA. We checked these for evidence of recombination using the programs described above.

### Estimation of substitution rates and divergence times

The phylogenetic relationships of the aligned full and partial genomic sequences were inferred using the Neighbor-Net method in SPLITSTREE v4.11.3^26^ and maximum likelihood in PhyML v3^45^. For the ML analysis, we used the general time-reversible (GTR) model of nucleotide substitution with rate variation among sites modeled using a gamma distribution and a proportion of invariable sites (GTR + I + G). This model was selected using jModelTest2^45, 46^. Branch support was evaluated by bootstrap analysis based on 1000 pseudoreplicates. The inferred trees were displayed using TreeView^47^.

The degree of mutational saturation in the aligned ORF sequences was evaluated using the Iss statistic in DAMBE^27^. BEAST v1.8.2^48^ was used to estimate the evolutionary rate and timescale of TuMV populations. Analyses were first based on complete sequences of the complete major ORF of the genomes (nt 131–9622, corresponding to the positions in the original TuMV-UK 1 isolate genome). Recombinant sequences were discarded from the ORF dataset (see Supplementary Table S2). The sampling times of the sequences were used to calibrate the molecular clock.

Bayes factors were used to select the best-fitting clock model and coalescent tree prior for each data set. We compared strict and relaxed (uncorrelated exponential and uncorrelated lognormal) clock models^28^, as well as five demographic models (constant population size, expansion growth, exponential growth, logistic growth and the Bayesian skyline plot). Posterior distributions of parameters, including the tree, were estimated by Markov chain Monte Carlo (MCMC) sampling. Samples were drawn every 10^4^ MCMC steps over a total of 10^8^ steps, with the first 10% of samples discarded as burn-in. Sufficient sampling from the posterior and convergence to the stationary distribution were checked using the diagnostic software Tracer v1.6 (http://tree.bio.ed.ac.uk/software/tracer/). Bayesian maximum-clade-credibility trees were generated with software included in the BEAST package.

For reliable rate estimates from time-structured sequence data, the range of sampling times needs to be wide enough to allow an appreciable amount of genetic change to occur^49, 50^. We checked the temporal signal in our data sets by comparing our rate estimates with those from ten date-randomized replicates. We used two different criteria to test for temporal structure, as described previously^29, 30^. According to the standard criterion, 95% CIs of date-randomized replicates should not overlap with the mean estimate from the original data set. A more conservative criterion, proposed by Duchêne *et al*.^30^, checks for overlap between the 95% CIs of the estimates from the date-randomized replicates and the original data set.

BEAST analyses were also done using the synonymous sites of TuMV polyprotein-encoding sequences. A simple pairwise sliding-window method DnDscan^51^ was used to identify codons in the alignments that had not evolved or had evolved non-synonymously. These codons were removed using SEQSPLIT v1.0 (written and provided by the late John Armstrong, http://192.55.98.146/_resources/e-texts/blobs/SeqSplit.ZIP). After silent sites were chosen from each protein region, those sequences were concatenated to produce 6078 nt sequences. The resulting sequences of the synonymous sites (300 to 6078 nt) of the major ORF, HC-Pro*, P3* and NIb* regions were 64%, 57%, 34% and 61% of the length of each complete protein-coding sequence (Table 1b). Non-synonymous (dN) and synonymous (dS) substitution (dN/dS) ratios were calculated using MEGA7^52^.

The spatial population dynamics of TuMV through time were inferred in BEAST using a diffusion model with discrete location states^33^. This approach uses a model that describes the spatial spread of TuMV lineages throughout their demographic history. The most important diffusions between pairs of locations can be identified using Bayes factors^53^. We produced a graphical animation of the estimated spatio-temporal movements of TuMV lineages using SPREAD v.1.0.6^54^ and Google Earth (http://www.google.com/earth).

The program MigraPhyla^34^ was used to infer the dissemination pathways of the virus. To estimate the reliability of the predicted dissemination events, a Monte Carlo simulation of 10 000 trials was performed by randomizing the character states of the leaf nodes while retaining the tree topologies. The sparse false discovery rate (sFDR) correction was used to account for multiple comparisons. Only the dissemination events with P < 0.05 and greater than the sFDR cut-off were considered significant. The dissemination pathways were represented using Circos^55, 56^ and marked on a map.

## Electronic supplementary material

Supplementary tables and figures
