# Supplementary material for: The Timescale of Emergence and Spread of Turnip Mosaic Potyvirus
Source: Sci Rep. 2017 Jun 26;7:4240. doi: 10.1038/s41598-017-01934-7 (PMC5484681; doi:10.1038/s41598-017-01934-7)
Supplement: Supplementary file 1 — Supplementary tables and figures [file 41598_2017_1934_MOESM1_ESM.pdf]

The Timescale of Emergence and Spread of Turnip Mosaic Potyvirus

Ryosuke Yasaka[1,2], Hirofumi Fukagawa[1], Mutsumi Ikematsu[1], Hiroko Soda[1], Savas Korkmaz[3], Alireza Golnaraghi[4], Nikolaos Katis[5], Simon Y. W. Ho[6], Adrian J. Gibbs[7] & Kazusato Ohshima[1,2]

[1]Laboratory of Plant Virology, Department of Applied Biological Sciences, Faculty of Agriculture, Saga University, 1-banchi, Honjo-machi, Saga 840-8502, Japan

[2]The United Graduate School of Agricultural Sciences, Kagoshima University, 1-21-24, Kagoshima, 890-0065, Japan

[3]Department of Plant Protection, Faculty of Agriculture, University of Canakkale Onsekiz Mart, Canakkale, Turkey

[4]Department of Plant Protection, College of Agriculture and Natural Resources, Science and Research Branch, Islamic Azad University, Tehran P.O. Box 14515-775, Iran

[5]Plant Pathology Laboratory, Faculty of Agriculture, Aristotle University of Thessaloniki, Thessaloniki 540 06, Greece

[6]School of Life and Environmental Sciences, University of Sydney, Sydney, NSW 2006, Australia

[7]Emeritus Faculty, Australian National University, Canberra, ACT 2601, Australia

Correspondence and requests for materials should be addressed to K.O. (ohshimak@cc.saga-u.ac.jp)

Supplementary Table S1. Turnip mosaic virus isolates analysed in this study.

Isolate	Original host	Location (City, district)	Year of collection	Host type[a]	Reference	Accession code
Greece						
GK1	*Matthiola incana*	Not known	<1989	B	1	AB701696
GRC 2	*Brassica oleracea* var. *capitata*	-, Thessaloniki	1993	B	This study	AP017816
GRC 3	*B. oleracea* var. *capitata*	-, Thessaloniki	1993	B	This study	AP017817
GRC 4	*B. oleracea* var. *capitata*	-, Thessaloniki	1993	B	This study	AP017818
GRC 5	*B. oleracea* var. *capitata*	-, Karvali	1993	B	This study	AP017819
GRC 6	*B. oleracea* var. *botrytis*	-, Karvali	1993	B	This study	AP017820
GRC 10	*B. oleracea* var. *botrytis*	-, Thessaloniki	1993	B	This study	AP017821
GRC 11	*B. oleracea* var. *botrytis*	-, Thessaloniki	1993	B	This study	AP017822
GRC 12	*B. oleracea* var. *botrytis*	-, Karvali	1993	B	This study	AP017823
GRC 16	*B. oleracea* var. *botrytis*	-, Karvali	1993	B(R)	This study	AP017824
GRC 17	*B. oleracea* var. *botrytis*	Volos, Magnesia	1993	B	2	AB252116
GRC 18	*B. oleracea* var. *botrytis*	-, Xanthi	1994	B	This study	AP017825
GRC 21	*B. oleracea* var. *capitata*	-, County Xanthi	1993	B	This study	AP017826
GRC 23	*B. oleracea* var. *capitata*	-, Thessaloniki	1993	B	This study	AP017827
GRC 24	*B. oleracea* var. *botrytis*	-, Thessaloniki	1993	B(R)	This study	AP017828
GRC 25	*B. oleracea* var. *botrytis*	-, Thessaloniki	1993	B	This study	AP017829
GRC 26	*B. oleracea* var. *botrytis*	-, Karvali	1993	B	This study	AP017830
GRC 27	*B. oleracea* var. *capitata*	-, County Xanthi	1993	B(R)	This study	AP017831
GRC 28	*B. oleracea* var. *capitata*	-, County Xanthi	1993	B	This study	AP017832
GRC 30	*B. oleracea* var. *capitata*	-, County Xanthi	1993	B	This study	AP017833
GRC 31	*B. oleracea* var. *botrytis*	Tenedos, Chalkidiki	1994	B	This study	AP017834
GRC 32	*B. oleracea* var. *botrytis*	-, Orestiada	1994	B	This study	AP017835
GRC 33	*B. oleracea* var. *capitata*	-, Orestiada	1994	B(R)	This study	AP017836
GRC 34	*B. oleracea* var. *capitata*	-, Lamia	1994	B	This study	AP017837
GRC 35	*B. oleracea* var. *botrytis*	-, Lamia	1993	B	This study	AP017838
GRC 37	*Brassica rapa*	-, Chalkidiki	1993	B(R)	This study	AP017715
GRC 38	*B. oleracea* var. *capitata*	Not known	1996	B(R)	This study	AP017839
GRC 39	*B. oleracea* var. *capitata*	Not known	1996	B	This study	AP017840
GRC 41	*Cheiranthus* sp.	Not known	1999	B	This study	AP017755
GRC 42	wild *Allium* sp.	Not known	1999	B	2	AB252117
GRC 43	wild *Allium* sp.	Not known	1999	B	This study	AP017756
GRC 44	*Allium hirsutum*	Not known	2000	B	This study	AP017716
GRC 45	*Allium neapolitanum*	Not known	2000	B	This study	AP017717
GRC 51	*Eruca sativa*	Neamagnisia, Thessaloniki	2007	B	This study	AP017713
GRC 53	*E. sativa*	Neamagnisia, Thessaloniki	2007	B(R)	This study	AP017718
GRC 55	*E. sativa*	Neamagnisia, Thessaloniki	2007	B	This study	AP017714
GRC 58	*E. sativa*	Neamagnisia, Thessaloniki	2007	B(R)	This study	AP017719
GRC 59	*Sinapis arvensis*	Achaia, Patra	2007	ND	This study	AP017720
GRC 60	*E. sativa*	-, Evia	2007	B	This study	AP017721
GRC 61	*E. sativa*	-, Evia	2007	B	This study	AP017722
GRC 62	*E. sativa*	-, Evia	2007	B	This study	AP017723
GRC 63	*E. sativa*	-, Evia	2007	B	This study	AP017724
GRC 65	*S. arvensis*	-, Evia	2007	ND	This study	AP017725
GRC 69	*S. arvensis*	Achaia, Patra	2007	B	This study	AP017712
GRC 70	*S. arvensis*	Achaia, Patra	2007	ND	This study	AP017726
GRC 71	*S. arvensis*	Achaia, Patra	2007	B	This study	AP017727
Iran						
IRN BRE2	*Raphanus sativus*	Falavarjan, Esfahan	2007	B(R)	This study	AP017841
IRN BRE4	*R. sativus*	Falavarjan, Esfahan	2007	B	This study	AP017757
IRN BRE7	*R. sativus*	Falavarjan, Esfahan	2007	BR	This study	AP017758
IRN BRE12	*R. sativus*	Falavarjan, Esfahan	2007	BR	This study	AP017759
IRN BRE19	*R. sativus*	Falavarjan, Esfahan	2007	B(R)	This study	AP017842
IRN BRSh12	*R. sativus*	Shiraz, Fars	2007	B(R)	This study	AP017843
IRN CM1	*Mattiola* sp.	Mahallat, Markazi	2003	B(R)	This study	AP017760
IRN CnQ	*Brassica napus*	Qom, Qom	2006	B(R)	This study	AP017761
IRN CQ	*Cheiranthus cheiri*	Qom, Qom	2003	B(R)	This study	AP017762
IRN CV1	*Mattiola* sp.	Varamin, Tehran	2003	B(R)	This study	AP017752
IRN CV2	*Mattiola* sp.	Varamin, Tehran	2003	B(R)	This study	AP017753
IRN DM	*Chrysanthemum* sp.	Mahallat, Markazi	2004	B(R)	This study	AP017763
IRN EAM1	*Mattiola* sp.	Tabiz, Azarbayjan-e-Sharqi	2005	B	This study	AP017754
IRN EB1	*B. oleracea* var. *italica*	Esfahan, Esfahan	2003	B	This study	AP017764
IRN EM1	*Mattiola* sp.	Esfahan, Esfahan	2004	B(R)	This study	AP017765
IRN ER1	*R. sativus*	Esfahan, Esfahan	2004	B(R)	This study	AP017766
IRN ES1	*R. sativus*	Esfahan, Esfahan	2003	B	This study	AP017767
IRN KBS58	*Sisymbrium loeselii*	Baft, Kerman	2006	B(R)	This study	AP017768
IRN KBS65	*S. loeselii*	Baft, Kerman	2006	B(R)	This study	AP017769
IRN KhCa	*B. oleracea* var. *botrytis*	Mashhad, Tehran	2005	B(R)	This study	AP017770
IRN M14	*B. rapa*	Mahallat, Markazi	2004	B	This study	AP017771
IRN M15	*B. rapa*	Mahallat, Markazi	2004	B(R)	This study	AP017772
IRN MB6	*B. rapa*	Mahallat, Markazi	2003	B	This study	AP017773
IRN MEr1	*E. sativa*	Mahallat, Markazi	2006	B(R)	This study	AP017774
IRN MEr3	*E. sativa*	Mahallat, Markazi	2006	B(R)	This study	AP017775
IRN MEr5	*E. sativa*	Mahallat, Markazi	2006	B(R)	This study	AP017776
IRN MM1	*Mattiola* sp.	Mahallat, Markazi	2003	B(R)	This study	AP017777
IRN MY57	*B. rapa*	Meybod, Yazd	2004	B	This study	AP017778
IRN RafCh	*C. cheiri*	Rafsanjan, Kerman	2006	B	This study	AP017779
IRN RaNi3	*Rapistrum rugosum*	Khorasan, Tehran	2005	B	This study	AP017780
IRN REY4	*R. sativus*	Esfahan, Esfahan	2004	B(R)	This study	AP017781
IRN RGhE	*R. sativus*	Ghahderijan, Esfahan	2005	B(R)	This study	AP017782
IRN RK	*R. rugosum*	Karaj, Tehran	2004	B	This study	AP017783
IRN Rkaraj	*R. rugosum*	Karaj, Tehran	2004	B(R)	This study	AP017784
IRN RN6	*R. rugosum*	Khorasan, Tehran	2005	B	This study	AP017785
IRN RRSh30	*R. sativus*	Shiraz, Fars	2007	B(R)	This study	AP017844

[a]Host type B; *Brassica*, isolates infected *B. rapa* cv. Hakatasuwari systemically giving mosaic symptoms. Host type (B); isolates infected *B. rapa* only occasionally. Host type BR; these isolates infected both *B. rapa* and *R. sativus* systemically giving mosaic symptoms. Host type B(R); isolates infected *B. rapa* systemically giving mosaic symptoms and infected *R. sativus* only occasionally.

[b]DI, difficult to infect brassica plant[1]

[c]Unclear

Supplementary Table S1. Continued.

Isolate	Original host	Location (City, district)	Year of collection	Host type	Reference	Accession code
IRN RRSh35	*R. sativus*	Shiraz, Fars	2007	B(R)	This study	AP017845
IRN RS2	*R. sativus*	Shiraz, Fars	2005	BR	This study	AP017786
IRN RS3	*R. sativus*	Shiraz, Fars	2005	B	This study	AP017787
IRN S1	*S. loeselii*	Semnan, Semnan	2003	B	This study	AP017788
IRN S3	*S. loeselii*	Semnan, Semnan	2003	B	This study	AP017789
IRN S4	*S. loeselii*	Semnan, Semnan	2003	B	This study	AP017790
IRN SA	*S. loeselii*	Absard, Tehran	2005	B	This study	AP017791
IRN ShA	*B. oleracea*	Shiraz, Fars	2003	B	This study	AP017792
IRN SRSh35	*R. sativus*	Shiraz, Fars	2007	BR	This study	AP017846
IRN SRSh37	*R. sativus*	Shiraz, Fars	2007	B(R)	This study	AP017793
IRN SS5	*S. loeselii*	Semnan, Semnan	2003	B	3	AB440239
IRN ST	*S. loeselii*	Tehran, Tehran	2005	B(R)	This study	AP017794
IRN TH	*Hirschfeldia incana*	Tehran, Tehran	2006	B	This study	AP017795
IRN TIm1	*Impatiens balsamina*	Varamin, Tehran	2004	B	This study	AP017796
IRN TKE	*Sisymbrium irio*	Karaj, Tehran	2006	B	This study	AP017797
IRN TM5	*Mattiola* sp.	Mahallat, Markazi	2003	B(R)	This study	AP017798
IRN TOF2	*S. loeselii*	Fasham, Tehran	2006	ND	This study	AP017799
IRN TOF3	*S. loeselii*	Fasham, Tehran	2006	B(R)	This study	AP017800
IRN TOF6	*S. loeselii*	Fasham, Tehran	2006	B(R)	This study	AP017801
IRN TP1	*Petunia hybrida*	Not known	2004	B	This study	AP017802
IRN TRa6	*R. rugosum*	Varamin, Tehran	2004	B	3	AB440238
IRN TRa9	*R. rugosum*	Varamin, Tehran	2004	B	This study	AP017803
IRN TRT9	*B. rapa*	Shahr-e-Rey, Tehran	2006	B	This study	AP017804
IRN TSh8	*B. rapa*	Shemiranat, Tehran	2006	B(R)	This study	AP017805
IRN TuK33	*R. sativus*	Karaj, Tehran	2004	B(R)	This study	AP017806
IRN TuM5	*B. rapa*	Mahallat, Markazi	2006	B(R)	This study	AP017807
IRN TuSh18	*B. rapa*	Shiraz, Fars	2007	B	This study	AP017808
IRN WRN8	*R. sativus*	Langarud, Gilan	2008	B(R)	This study	AP017847
IRN WRN9	*R. sativus*	Langarud, Gilan	2008	BR	This study	AP017848
IRN WRN10	*R. sativus*	Langarud, Gilan	2008	B(R)	This study	AP017849
IRN WRN11	*R. sativus*	Langarud, Gilan	2008	BR	This study	AP017850
IRN WRSh1	*R. sativus*	Shiraz, Fars	2007	B(R)	This study	AP017851
IRN WRSh10	*R. sativus*	Shiraz, Fars	2007	B(R)	This study	AP017852
IRN WRSh12	*R. sativus*	Shiraz, Fars	2007	BR	This study	AP017809
IRN WRSh20	*R. sativus*	Shiraz, Fars	2007	BR	This study	AP017810
IRN WRSh24	*R. sativus*	Shiraz, Fars	2007	BR	This study	AP017853
IRN WRSh32	*R. sativus*	Shiraz, Fars	2007	BR	This study	AP017811
IRN WRSh40	*R. sativus*	Shiraz, Fars	2007	B(R)	This study	AP017854
IRN WRSh42	*R. sativus*	Shiraz, Fars	2007	BR	This study	AP017855
IRN WRSh203	*R. sativus*	Shiraz, Fars	2008	BR	This study	AP017856
IRN WRWA5	*R. sativus*	Orumiyeh, Azarbayjan-e-Gharbi	2008	B(R)	This study	AP017812
IRN WRWA14	*R. sativus*	Orumiyeh, Azarbayjan-e-Gharbi	2008	B(R)	This study	AP017857
IRN ZE	*Zinia elagans*	Esfahan, Esfahan	2007	BR	This study	AP017813
Turkey						
TUR1	*B. oleracea* var. *capitata*	-, Canakkale	2004	B	4	AB362512
TUR3	*B. oleracea* var. *capitata*	-, Canakkale	2004	B(R)	This study	AP017858
TUR4	*B. oleracea* var. *capitata*	-, Balikesir	2005	B	This study	AP017859
TUR5	*B. oleracea* var. *capitata*	-, Canakkale	2005	B(R)	This study	AP017860
TUR6	*B. oleracea* var. *capitata*	-, Canakkale	2005	B(R)	This study	AP017861
TUR7	*B. oleracea* var. *capitata*	-, Canakkale	2005	B(R)	This study	AP017862
TUR8	*B. oleracea* var. *gemmifera*	-, Canakkale	2005	B	This study	AP017863
TUR9	*R. sativus*	-, Balikesir	2005	BR	4	AB362513
TUR10	*R. sativus*	-, Balikesir	2006	BR	This study	AP017864
TUR13	*R. sativus*	-, Bursa	2006	BR	This study	AP017865
TUR14	*R. sativus*	-, Bursa	2006	BR	This study	AP017866
TUR16	*Raphanus raphanistrum*	-, Canakkale	2006	B(R)	This study	AP017728
TUR19	*R. raphanistrum*	-, Canakkale	2006	B	This study	AP017729
TUR20	*R. raphanistrum*	Center, Canakkale	2006	B(R)	This study	AP017867
TUR25	*E. sativa*	Edremit, Balikesir	2006	B	This study	AP017730
TUR27	*R. raphanistrum*	Karacabey, Bursa	2006	BR	This study	AP017868
TUR30	*Brassica* sp.	Cay, Afyon	2006	B	This study	AP017869
TUR31	*R. raphanistrum*	Sultandagi, Afyon	2006	B	This study	AP017870
TUR34	*R. raphanistrum*	Center, Hatay	2006	B(R)	This study	AP017731
TUR36	*R. raphanistrum*	Tarsus, Mersin	2006	B	This study	AP017871
TUR40	*B. rapa*	Nazilli, Aydin	2006	B	This study	AP017732
TUR41	*B. rapa*	Nazilli, Aydin	2006	B(R)	This study	AP017733
TUR42	*R. raphanistrum*	Nazilli, Aydin	2006	B(R)	This study	AP017734
TUR43	*B. rapa*	Nazilli, Aydin	2006	B	This study	AP017735
TUR44	*R. raphanistrum*	Nazilli, Aydin	2006	B	This study	AP017736
TUR45	*B. rapa*	Sultanhisar, Aydin	2006	B	This study	AP017737
TUR47	*E. sativa*	Sultanhisar, Aydin	2006	B	This study	AP017738
TUR49	*B. rapa*	Torbali, Izmir	2006	B	This study	AP017872
TUR50	*B. rapa*	Torbali, Izmir	2006	B	This study	AP017739
TUR51	*E. sativa*	Center, Canakkale	2006	B	This study	AP017740
TUR52	*S. arvensis*	Center, Canakkale	2006	B(R)	This study	AP017873
TUR55	*E. sativa*	Cumra, Konya	2007	B	This study	AP017741
TUR56	*B. rapa*	Cumra, Konya	2007	B	This study	AP017874
TUR57	*B. rapa*	Cumra, Konya	2007	B(R)	This study	AP017742
TUR58	*B. oleracea* var. *capitata*	-, Konya	2007	B(R)	This study	AP017875
TUR59	*R. raphanistrum*	-, Konya	2007	B	This study	AP017743
TUR60	*R. raphanistrum*	-, Konya	2007	B	This study	AP017744
TUR61	*R. raphanistrum*	-, Konya	2007	ND	This study	AP017745
TUR62	*B. oleracea* var. *capitata*	-, Konya	2007	B	This study	AP017876
TUR63	*R. raphanistrum*	-, Nigde	2007	B	This study	AP017877
TUR64	*R. raphanistrum*	-, Nigde	2007	B	This study	AP017878
TUR65	*B. oleracea* var. *capitata*	-, Nigde	2007	B(R)	This study	AP017879
TUR66	*B. oleracea* var. *capitata*	-, Nigde	2007	B(R)	This study	AP017880
TUR67	*B. oleracea* var. *capitata*	-, Nigde	2007	B(R)	This study	AP017881
TUR68	*B. oleracea* var. *capitata*	-, Nigde	2007	B(R)	This study	AP017882
TUR73	*B. oleracea* var. *botrytis*	Inegol, Bursa	2007	B(R)	This study	AP017883
TUR77	*R. sativus*	-, Canakkale	2007	BR	This study	AP017884

Supplementary Table S1. Continued.

Isolate	Original host	Location (City, district)	Year of collection	Host type	Reference	Accession code
TUR78	*R. raphanistrum*	-, Canakkale	2007	B	This study	AP017746
TUR79	*R. raphanistrum*	Yakapinar, Adana	2007	B	This study	AP017814
TUR80	*B. oleracea* var. *capitata*	Havutlu, Adana	2007	B(R)	This study	AP017885
TUR84	*R. raphanistrum*	-, Urfa	2007	B(R)	This study	AP017886
TUR85	*R. raphanistrum*	-, Urfa	2007	B(R)	This study	AP017887
TUR86	*R. raphanistrum*	-, Urfa	2007	B(R)	This study	AP017888
TUR88	*R. raphanistrum*	Birecik, Urfa	2007	B(R)	This study	AP017747
TUR90	*R. raphanistrum*	Birecik, Urfa	2007	B	This study	AP017748
TUR91	Not known	-, Ankara	2007	B	This study	AP017889
TUR95	*E. sativa*	Dikili, Izmir	2007	B(R)	This study	AP017749
TUR97	*R. raphanistrum*	Bayramic, Canakkale	2007	B(R)	This study	AP017750
TUR106	*B. oleracea* var. *capitata*	Tekkekoy, Samsun	2008	B(R)	This study	AP017890
TUR242	*E. sativa*	-, Bolu	2012	B(R)	This study	AP017815
TUR244	*Spinacia oleracea*	-, Bolu	2012	B(R)	This study	AP017751
Asia						
China						
BJ-B01	*B. oleracea* var. *capitata*	Beijing, Beijing	2010	Not known		KC119185
BJ-B02	*B. oleracea* var. *capitata*	Beijing, Beijing	2010	Not known		KC119186
BJ-B03	*B. oleracea* var. *capitata*	Beijing, Beijing	2010	Not known		KC119187
BJ-B04	*B. oleracea* var. *capitata*	Beijing, Beijing	2009	Not known		KC119188
BJ-B05	*B. oleracea* var. *capitata*	Beijing, Beijing	2009	Not known		KC119189
BJ-C4	*B. oleracea* var. *capitata*	Beijing, Beijing	1985-1987	Not known		HQ446217
BJ-R01	*R. sativus*	Beijing, Beijing	2010	Not known		KC119184
CH6	*R. sativus*	Zengjiang, Jiangsu	1999	BR	2	AB252103
CHK16	*R. sativus*	Guilin, Guangxi	2000	BR	2	AB252104
CHL13	*R. sativus*	Lushun, Liaoning	1999	BR	2	AB252105
CHN 12	Not known	Not known	<1990	B	5	AY090660
CHZJ26A	*Brassica campestris*	Jiande, Zhejiang	1999	B(R)	2	AB252106
HRD	*R. sativus*	Hongzhou, Zhejiang	1998	BR	6	AB093627
HZ6	*Brassica* sp.	Xiaoshan, Zhejiang	1998	B	2	AB252119
Lu2	*Brassica* sp.	-, Shandong	1986-1990	Not known		HQ446216
TANX2	*R. sativus*	Tai'an, Shandong	2007	BR	7	EU734433
WFLB06	*R. sativus*	Weifang, Shandong	2006	BR	7	EU734434
ZH1	*Phalaenopsis* sp.	Not known	2012	Not known		KF246570
Israel						
IS1	*Allium ampeloprasum*	Not known	1993	B	6	AB093602
Japan						
1J	*R. sativus*	Saga, Saga	1977	BR	8	D83184
2J	*Brassica pekinensis*	-, Tochigi	1994	BR	6	AB093622
59J	*R. sativus*	Saga, Saga	1996	BR	6	AB093620
AD178J	*R. sativus*	Rokunohe, Aomori	1998	BR	2	AB252094
AD181J	*R. sativus*	Tohoku, Aomori	1998	BR	2	AB252095
AD853J	*R. sativus*	Ohhata, Aomori	2002	BR	2	AB252096
AD855J	*R. sativus*	Ohminato, Aomori	2002	BR	2	AB252097
AD860J	*R. sativus*	Sennai, Aomori	2002	BR	2	AB252098
AKD161J	*R. sativus*	Ogachi, Akita	1998	BR	2	AB252099
AKD934J	*R. sativus*	Hachiryu, Akita	2000	BR	2	AB252100
AKH937J	*B. pekinensis*	Yuzawa, Akita	2000	BR	2	AB252101
AT181J	*Eustoma russellianum*	Aomori, Aomori	<1998	BR	2	AB252102
C42J	*B. rapa*	Saga, Saga	1993	B	6	AB093625
CP845J	*Calendula officinalis*	Kisarazu, Chiba	1997	BR	6	AB093614
DMJ	*R. sativus*	-, Tochigi	1996	BR	6	AB093623
FD27J	*R. sativus*	Fukuoka, Fukuoka	1998	BR	6	AB093618
FKD001J	*R. sativus*	Sukagawa, Fukushima	2000	BR	2	AB252109
FKD004J	*R. sativus*	Funehiki, Fukushima	2000	BR	2	AB252110
FKH122J	*B. pekinensis*	Naraha, Fukushima	1998	BR	2	AB252111
GFD462J	*R. sativus*	Yoro, Gifu	2001	BR	2	AB252115
H1J	*R. sativus*	Hirosaki, Aomori	1996	BR	2	AB252118
HOD517J	*R. sativus*	Kimobetsu, Hokkaido	1998	BR	6	AB093617
IWD032J	*R. sativus*	Iwaizumi, Iwate	2000	BR	2	AB252120
IWD038J	*R. sativus*	Yahaba, Iwate	2000	BR	2	AB252121
JPN1	*R. sativus*	Not known	1995	Not known		KM094174
Ka1J	*B. pekinensis*	-, Tochigi	1994	BR	6	AB093624
KD32J	*R. sativus*	Nankan, Kumamoto	1998	BR	6	AB093621
KGD54J	*R. sativus*	Sendai, Kagoshima	1998	BR	2	AB252123
KWB778J	*B. oleracea* var. *botrytis*	Takamatsu, Kagawa	2004	B	2	AB252124
KWB779J	*B. rapa*	Takamatsu, Kagawa	2004	BR	2	AB252125
KYD073J	*R. sativus*	Mineyama, Kyoto	2000	BR	2	AB252126
KYD81J	*R. sativus*	Joyo, Kyoto	1998	BR	6	AB093613
MED302J	*R. sativus*	Shiroyama, Mie	2001	BR	2	AB252127
MYD013J	*R. sativus*	Yamamoto, Miyagi	2000	BR	2	AB252128
MYD015J	*R. sativus*	Kesennuma, Miyagi	2000	BR	2	AB252129
ND10J	*R. sativus*	Hirato, Nagasaki	1998	BR	2	AB252130
NDJ	*R. sativus*	Takaki, Nagasaki	1997	BR	6	AB093616
NID048J	*R. sativus*	Niitsu, Niigata	2000	BR	2	AB252131
NID119J	*R. sativus*	Yuzawa, Niigata	1998	BR	2	AB252132
NRD350J	*R. sativus*	Gojyo, Nara	2001	BR	2	AB252134
SGB088J	*B. rapa*	Hikone, Shiga	2000	BR	2	AB252136
SGD311J	*R. sativus*	Nishiazai, Shiga	1998	BR	6	AB093619
SMD060J	*R. sativus*	Gotsu, Shimane	2000	BR	2	AB252137
TD88J	*R. sativus*	Tokyo, Tokyo	1998	BR	6	AB093615
TRD052J	*R. sativus*	Akasaki, Tottori	2000	BR	2	AB252138
TRD053J	*R. sativus*	Tomari, Tottori	2000	BR	2	AB252139
Tu-2R1	*R. sativus*	-, Tochigi	Not known	BR	9	AB105135
Tu-3	*B. oleracea* var. *capitata*	-, Tochigi	Not known	B	9	AB105134
YAD020J	*R. sativus*	Shirataka, Yamagata	2000	BR	2	AB252140
YAL018J	*Lactuca sativa*	Sakae, Yamagata	2000	BR	2	AB252141
YMD069J	*R. sativus*	Misumi, Yamaguchi	2000	BR	2	AB252142
YMD070J	*R. sativus*	Abu, Yamaguchi	2000	BR	2	AB252143

Supplementary Table S1. Continued.

Isolate	Original host	Location (City, district)	Year of collection	Host type	Reference	Accession code
Taiwan						
C1	Not known	Not known	Not known	Not known		AF394601
CHN 1	*Brassica* sp.	Not known	<1980	BR	6	AB093626
RC4	*Zantedeschia* sp.	Not known	2000	BR	10	AY134473
TW	Not known	Not known	Not known	Not known		AF394602
YC5	*Zantedeschia* sp.	Not known	2000	BR	10	AF530055
Vietnam						
VIET15	*R. sativus*	Van Giang, Hung Yen	2006	B(R)	11	AB747286
VIET56	*Brassica juncea*	Moc Chau, Son La	2007	B	11	AB747287
VIET58	*B. juncea*	Moc Chau, Son La	2007	BR	11	AB747288
VIET65	*R. sativus*	Gia Lam, Ha Noi	2007	BR	11	AB747289
VIET66	*R. sativus*	Gia Lam, Ha Noi	2007	B	11	AB747290
VIET73	*R. sativus*	Van Giang, Hung Yen	2007	BR	11	AB747291
VIET79	*R. sativus*	Cam Giang, Hai Dung	2007	BR	11	AB747292
VIET80	*R. sativus*	Cam Giang, Hai Dung	2007	B	11	AB747293
VIET82	*R. sativus*	Ban Me Thuot, Dak Lak	2007	B(R)	11	AB747294
VIET83	*R. sativus*	Ban Me Thuot, Dak Lak	2007	B	11	AB747295
VIET89	*R. sativus*	Ban Me Thuot, Dak Lak	2007	BR	11	AB747296
VIET138	*B. juncea*	Thanh Long, Thua Thien Hue	2007	B(R)	11	AB747297
VIET153	*B. juncea*	Hoi An, Quang Nam	2007	B(R)	11	AB747298
VIET158	*B. juncea*	Gia Lam, Ha Noi	2007	B(R)	11	AB747299
VIET159	*B. juncea*	-, Lang Son	2007	B	11	AB747300
VIET160	*B. juncea*	Huu Lung, Lang Son	2007	B	11	AB747301
VIET164	*B. juncea*	Thuong Tin, Ha Tay	2007	B	11	AB747302
VIET166	*B. juncea*	Thuong Tin, Ha Tay	2007	B	11	AB747303
VIET167	*B. juncea*	Gia Lam, Ha Noi	2007	B	11	AB747304
VIET169	*B. juncea*	Vo Cuong, Bac Ninh	2007	B	11	AB747305
VIET170	*B. juncea*	Vo Cuong, Bac Ninh	2007	B(R)	11	AB747306
VIET172	*B. juncea*	Gia Lam, Ha Noi	2007	B	11	AB747307
VIET173	*B. juncea*	Viet Yen, Bac Giang	2007	B	11	AB747308
VIET174	*B. juncea*	Viet Yen, Bac Giang	2007	B	11	AB747309
VIET175	*B. juncea*	Viet Yen, Bac Giang	2007	B(R)	11	AB747310
VIET176	*B. juncea*	Vu Thu, Thai Binh	2007	B(R)	11	AB747311
VIET177	*B. juncea*	Vu Thu, Thai Binh	2008	B	11	AB747312
VIET178	*B. juncea*	Nam Truc, Nam Dinh	2008	B(R)	11	AB747313
VIET179	*B. juncea*	Nam Truc, Nam Dinh	2008	B(R)	11	AB747314
VIET180	*B. juncea*	Viet Yen, Bac Giang	2008	B(R)	11	AB747315
Europe						
Denmark						
AllA	*Alliaria officinalis*	Not known	1991	B	1	AB701694
DNK 2	*B. napus*	Not known	<1993	B	2	AB252108
DNK 3	*B. rapa*	Not known	1978	B	2	AB701703
DNK 4	*B. rapa*	Not known	1986	B	1	AB701704
Germany						
ASP	*Allium* sp.	Gatersleben, Sachsen-Anhalt	1995	B	1	AB701697
DEU 1	Not known	Not known	<1976	B	1	AB701699
DEU 2	*R. sativus*	Not known	<1993	B	1	AB701700
DEU 4	*L. sativa*	Stuttgart, Baden-Württemberg	1986	BR	1	AB701701
DEU 5	*L. sativa*	Monchengladbach, Nordrhein-Westfalen	1991	B	1	AB701702
DEU 7	*L. sativa*	Frankfurt, Hessen	1994	B	1	AB701695
FRD 1	*B. oleracea* var. *capitata*	Not known	1987	B	2	AB252112
OM-A	*Orchis militaris*	Celle, Lower Saxony	1981	DI[b]	1	AB701691
OM-N	*O. militaris*	Celle, Lower Saxony	1981	DI	1	AB701690
ORM	*Orchis morio*	Celle, Lower Saxony	1983	(B)	1	AB701692
OS	*Orchis simia*	Celle, Lower Saxony	1981	DI	1	AB701693
PV0054	*B. oleracea*	Not known	Not known	B	1	AB701730
PV0104	*L. sativa*	Stuttgart, Baden-Württemberg	1986	BR	6	AB093603
PV376-Br	*B. napus*	Braunschweig, Lower Saxony	1970	B	6	AB093604
TIGA	*Tigridia* sp.	Braunschweig, Lower Saxony	1983	(B)	1	AB701734
TIGD	*Tigridia* sp.	Braunschweig, Lower Saxony	1983	(B)	1	AB701735
UT	*Utricularia* sp.	Wuerzburg, Bayern	1997	B	1	AB701736
Italy						
A102/11	*Anemone coronaria*	-, Liguria	1993	B	6	AB093597
A64	*A. coronaria*	-, Liguria	1991	B	6	AB093599
Al	*A. officinalis*	-, Piedmont	1968	(B)	6	AB093598
Cal1	*C. officinalis*	-, Liguria	1979	BR	6	AB093601
Eru 1D	*E. sativa*	-, Piedmont	1991	B	1	AB701705
ITA 1A	*B. ruvo*	-, Campania	1990	B	1	AB701720
ITA 2	*C. cheiri*	-, Campania	1992	B	1	AB701721
ITA 3	*Brassica ruvo*	-, Campania	1990	B	2	AB252122
ITA 4	*B. rapa*	-, Campania	1990	B	1	AB701722
ITA 5	*B. ruvo*	-, Campania	1990	B	1	AB701723
ITA 6	*M. incana*	-, Campania	1992	B	1	AB701724
ITA 7	*R. raphanistrum*	-, Campania	1990	BR	6	AB093600
ITA 8	*Abutilon* sp.	-, Campania	Sept.1993	BR	1	AB701725
ITA 9A	*Cucurbita pepo*	Not known	<1995	B	1	AB701726
Rn98	*Ranunculus asiaticus*	-, Liguria	1997	B	2	AB252135
St48	*Limonium sinuatum*	-, Toscana	1993	B	6	AB093596
Poland						
CAR37	*Cochlearia armoracia*	Not known	2004	Not known	12	DQ648592
CAR37A	*C. armoracia*	Not known	2004	Not known	12	DQ648591
CAR39	*C. armoracia*	Not known	2004	Not known		EF374098
CAR51	*C. armoracia*	Not known	2004	Not known		HQ637383
POL 1	*B. napus oleifera*	Poznan, Wielkopolska	<Oct.1993	B	1	AB701728
POL 2	*Papaver somniferum*	Czempin, -	<Oct.1993	B	1	AB701731
POL 4	*B. napus oleifera*	Grabianowo, Wielkopolska	<Oct.1993	B	1	AB701732

Supplementary Table S1. Continued.

Isolate	Original host	Location (City, district)	Year of collection	Host type	Reference	Accession code
Russia						
I2	*Brassica* sp.	Moscow, Moscow	Not known	Not known	13	KC297103
RUS 1	*Armoracia rusticana*	Not known	1993	B	6	AB093606
RUS 2	*B. napus*	Moscow, Moscow	Not known	B	6	AB093607
UK						
GBR 7	*Rheum rhabarbarum*	-, Gloucestershire	Sept.1993	B	1	AB701709
GBR 8	*Lunaria annua*	-, Essex	Apr.1994	B	1	AB701710
GBR 27	wild *B. oleracea*	Kimmeridge, Dorset	Mar.1999	B	1	AB701711
GBR 30	wild *B. oleracea*	Kimmeridge, Dorset	Apr.1999	B	1	AB701712
GBR 31	wild *B. oleracea*	Chapman's Pool, Dorset	Apr.1999	B	1	AB701713
GBR 32	wild *B. oleracea*	Chapman's Pool, Dorset	Apr.1999	B	1	AB701714
GBR 36	wild *B. oleracea*	Winspit, Dorset	Jun.1999	B	2	AB252113
GBR 38	wild *B. oleracea*	Winspit, Dorset	Jul.1999	B	1	AB701715
GBR 50	wild *B. oleracea*	Staithes, Yorkshire	Sept.1999	B	2	AB252114
GBR 51	wild *B. oleracea*	Staithes, Yorkshire	Sept.1999	B	1	AB701742
GBR 57	wild *B. oleracea*	Llandudno, Conwy	Sept.2000	B	1	AB701716
GBR 83	wild *B. oleracea*	Llandudno, Conwy	Aug.2002	B	1	AB701717
GBR 91	wild *B. oleracea*	Llandudno, Conwy	Aug.2002	B	1	AB701718
GBR 98	wild *B. oleracea*	Winspit, Dorset	Aug.2002	B	14	EU861593
PV177	*Brassica* spp.	Cambridge, Cambridgeshire	1934[c]	B	1	AB701733
UK 1	*B. napus*	-, Warwickshire	1975	B	15	AF169561
Other Europe						
BEL 1	*Rorippa nasturtium-aquaticum*	Wavre, Walloon Brabant, Belgium	1986	B	1	AB701698
CRO184A	*B. napus*	Zagreb, Zagreb, Croatia	May.2009	Not known	16	KF595121
CZE 1	*B. oleracea* var. *capitata*	Ruzyne-Prague, Prague, Czech Republic	1981	B	6	AB093608
CZE 5	*B. rapa*	Ceske Budejovice, South Bohemian Region, Czech Republic	1993	B	2	AB252107
ESP 1	*Eruca vesicaria* subsp. sativa	-, -, Spain	2001	B	1	AB701706
ESP 2	*Sisymbrium orientale*	Las Matas, Aragón, Spain	2001	B	1	AB701707
FRA 2	*B. napus*	-, -, France	<1994	B	1	AB701708
HUN 1	*Alliaria petiolata*	-, -, Hungary	<1996	B	1	AB701719
NLD 1	*B. oleracea* var. *gemmifera*	-, -, The Netherlands	<1995	B	2	AB252133
NLD 2	*B. oleracea*	-, -, The Netherlands	<1995	B	1	AB701727
PRT 1	*B. oleracea acephala*	-, Madeira, Portugal	1993/1994	B	1	AB701729
Oceania						
Australia						
AU1	*H. incana*	Canberra, ACT	1998	B	17	AB989628
AUST1 (3696F)	*Cicer arietinum*	Myall Vale-Narrabri, NSW	2003	B	17	AB989629
AUST2 (3834A)	*R. rugosum*	Breeza, NSW	2004	B(R)	17	AB989630
AUST3 (3896A)	*B. juncea*	Breeza, NSW	2004	B	17	AB989631
AUST4 (3896B)	*B. juncea*	Breeza, NSW	2004	B(R)	17	AB989632
AUST6 (Q1280)	*R. rugosum*	Allora, QLD	2001	B	17	AB989633
AUST10 (Q186)	*B. pekinensis*	Kalbar, QLD	1996	B	17	AB989634
AUST13 (Q484)	*B. pekinensis*	Gatton, QLD	1994	B	17	AB989635
AUST19 (Q2080)	*R. raphanistrum*	Melbourne, VIC	2007	B(R)	17	AB989636
AUST21 (5346A)	*H. incana*	Tamworth, NSW	2011	B	17	AB989637
AUST22 (5349A)	*R. rugosum*	Tamworth, NSW	2011	B	17	AB989638
AUST23 (Q2081)	*R. raphanistrum*	Melbourne, VIC	2007	B(R)	17	AB989639
AUST26 (4323A)	*B. rapa*	Mullaley, NSW	2007	B	17	AB989640
AUST27 (4323C)	*B. rapa*	Mullaley, NSW	2007	B	17	AB989641
AUST28 (5346B)	*H. incana*	Tamworth, NSW	2011	B	17	AB989642
AUST29 (5349B)	*R. rugosum*	Tamworth, NSW	2011	B	17	AB989643
BRS1	*B. rapa*	Brisbane, NSW	2007	Not known		HM544042
NSW1	*B. juncea*	-, NSW	2006	B	18	KJ936087
NSW2	*B. juncea*	-, NSW	2006	B	18	KJ936088
NSW3	*C. arietinum*	-, NSW	2006	B	18	KJ936089
NSW4	*R. rugosum*	-, NSW	2006	B	18	KJ936090
NSW5	*H. incana*	-, NSW	2006	B	18	KJ936091
NSW6	*R. rugosum*	-, NSW	2006	B	18	KJ936092
WA-Ap	*R. raphanistrum*	-, Western Australia	2000	B	18	KJ936093
New Zealand						
NZ11	*Crocus sativus*	Mid Canterbury-South Island	2002	Not known	17	AB989644
NZ12	*Nasturtium officinale*	Kumeu, Auckland-North Island	2003	Not known	17	AB989645
NZ246	*B. napus* cv. York Globe	Hornby, Mid Canterbury-South Island	1995	B	17	AB989646
NZ290	*B. pekinensis*	Hornby, Mid Canterbury-South Island	1998	B	6	AB093612
NZ402	*Lepidium oleraceum*	Stony Bay ,Banks Peninsula, South Island	2010	B	17	AB989647
NZ403	*L. oleraceum*	Island Rock, Banks Peninsula, South Island	2010	B	17	AB989657
NZ403B	*L. oleraceum*	Island Rock Banks Peninsula, South Island	2010	B	17	AB989658
NZ412	*Pachycladon fastigiatum*	Lincoln, Mid Canterbury-South Island	2010	B	17	AB989648
NZ412B	*P. fastigiatum*	Lincoln, Mid Canterbury-South Island	2010	B	17	AB989649
NZ415	*L. oleraceum*	Bridge Point,Otago,South Island	2010	B	17	AB989659
NZ419	*B. rapa* cv. Marco	Methven, Mid Canterbury-South Island	2010	B	17	AB989650
NZ419B	*B. rapa* cv. Marco	Methven, Mid Canterbury-South Island	2010	B	17	AB989651
NZ419C	*B. rapa* cv. Marco	Methven, Mid Canterbury-South Island	2010	B	17	AB989652
NZL5 (NZ298)	*Brassica* sp.	Lincoln, Canterbury-South Island	1999	B	17	AB989653
NZW3 (NZ299)	*B. rapa*	Lincoln, Mid Canterbury-South Island	1999	B	17	AB989654
NZW4 (NZ300)	*B. rapa*	Lincoln, Mid Canterbury-South Island	1999	B	17	AB989655
NZW6 (NZ302)	*B. rapa*	Lincoln, Mid Canterbury-South Island	1999	B	17	AB989656

Supplementary Table S1. Continued.

Isolate	Original host	Location (City, district)	Year of collection	Host type	Reference	Accession code
North America						
USA						
PV134	*Sesynibium* sp.	-, California	<1960	B	1	AB701737
PV389	*Tulipa gesnerana*	Beltsville, Maryland	1986	B	1	AB701738
USA1	*B. oleracea* var. *capitata*	Not known	<1980	B	6	AB093609
USA4	*B. pekinensis*	Not known	1993	B	1	AB701739
USA5	*R. sativus*	San Francisco, California	2002	BR	1	AB701740
USA6	*R. sativus*	San Francisco, California	2002	BR	1	AB701741
Canada						
CDN 1	*B. napus napobrassica*	Not known	<1988	B	15	AY227024
Q-Ca	*B. rapa*	Not known	Not known	Not known	19	D10927
South America						
Brazil						
BZ1	*B. oleracea* var. *acephala*	-, Federal	1996	B	6	AB093611
Africa						
Kenya						
KEN 1	*B. oleracea* var. *acephala*	Not known	1994	B	6	AB093605

Supplementary TableS2. Recombination sites in the genomes of turnip mosaic virus collected in Asian Minor and its neighboring countries.

Isolate	Nucleotide position and protein encoding region[a]	Parental isolate and subgroup[b]		Recombination detection program[c]	*P*-value[d]	*Z*-value[e]
		Major	Minor			
Greece						
GK1	1681 (HC-Pro)	GRC 44 (bB2)	GRC 41 (bB2)	**R**GBMCS$_R$S$_O$	1.92×10^{-36}	6.67
	5531 (CI) - 6094 (VPg)	GRC 44 (bB2)	GRC 41 (bB2)	RGBMC**S$_R$**S$_O$	1.51×10^{-21}	3.03, 3.51
	6222 (VPg)	GRC 45 (bB2)	TUR244 (bB2)	**R**BMCS$_R$S$_O$	1.40×10^{-17}	5.00
GRC 17	811 (P1)	CDN 1 (wB2)	YAD020J (wB3)	BC**S$_R$**S$_O$	1.04×10^{-15}	5.08
	5065 (CI)	QCa (wB2)	YAD020J (wB3)	RGBMC**S$_R$**	5.46×10^{-33}	2.14
	8921 (CP)	QCa (wB2)	YAD020J (wB3)	RBMC**S$_R$**	4.47×10^{-31}	-
GRC 18	7302 (NIb)	C1 (wB3)	TUR1 (wB2)	R**B**MCS$_R$S$_O$	1.25×10^{-13}	3.64
GRC 21	7302 (NIb)	C1 (wB3)	TUR1 (wB2)	R**B**MCS$_R$S$_O$	1.88×10^{-13}	3.64
GRC 27	494 (P1)	NZ11 (wB3)	TRD053J (ABR)	RG**B**MCS$_R$S$_O$	5.36×10^{-79}	7.12
	5125 (CI)	AT181J (wB3)	MYD015J (ABR)	**R**GBMCS$_R$S$_O$	2.96×10^{-139}	6.17
GRC 28	7302 (NIb)	C1 (wB3)	TUR1 (wB2)	R**B**MCS$_R$S$_O$	3.73×10^{-13}	3.89
GRC 30	7302 (NIb)	C1 (wB3)	TUR1 (wB2)	R**B**MCS$_R$S$_O$	1.84×10^{-13}	3.64
GRC 32	1737 (HC-Pro)	TUR62 (wB2)	GRC 31 (wB3)	**R**GBMCS$_R$S$_O$	1.75×10^{-53}	4.58
	2508 (HC-Pro) – 6277 (VPg)	QCa (wB2)	GRC 31 (wB3)	RGBMC**S$_R$**S$_O$	4.64×10^{-49}	3.02
GRC 33	1737 (HC-Pro)	TUR62 (wB2)	GRC 31 (wB3)	**R**GBMCS$_R$S$_O$	9.51×10^{-55}	4.05
	2508 (HC-Pro) – 6277 (VPg)	QCa (wB2)	GRC 31 (wB3)	RGBMC**S$_R$**S$_O$	4.87×10^{-49}	4.98
GRC 37	260 (P1)	GRC 65 (bB2)	TUR19 (bB2)	RGBMC**S$_R$**S$_O$	1.13×10^{-33}	4.18
	7120 (NIa-Pro)	GRC 45 (bB2)	TUR19 (bB2)	RGBMC**S$_R$**S$_O$	1.57×10^{-16}	6.23
	8963 (CP)	TUR242 (bB2)	TUR97 (bB2)	RGBMC**S$_R$**S$_O$	7.76×10^{-19}	4.02
GRC 41	706 (P1)	IRN TRT9 (bB2)	TUR52 (ABR)	RGBM**S$_R$**S$_O$	1.61×10^{-23}	5.27
GRC 44	423 (P1) – 1064 (P1)	TUR19 (bB2)	TUR86 (bB2)	**R**GBMCS$_R$S$_O$	1.11×10^{-39}	4.69
	2523 (HC-Pro) – 3327 (P3)	TUR19 (bB2)	TUR86 (bB2)	**R**GBMCS$_R$S$_O$	5.21×10^{-27}	4.57
GRC 45	775 (P1)	TUR91 (bB2)	TUR244 (bB2)	GBMC**S$_R$**	2.28×10^{-30}	2.90
	1167 (P1)	TUR91 (bB2)	TUR19 (bB2)	RGBMC**S$_R$**S$_O$	2.12×10^{-39}	3.68
	2076 (HC-Pro)	TUR244 (bB2)	GK1 (bB2)	RGBMC**S$_R$**S$_O$	1.47×10^{-40}	5.59
	7780 (NIb)	TUR242 (bB2)	TUR51 (bB2)	**R**GBMCS$_R$S$_O$	3.44×10^{-37}	7.02
	9130 (CP)	TUR57 (bB2)	TUR27 (ABR)	RGB**S$_R$**S$_O$	2.31×10^{-10}	4.05
GRC 51	7120 (NIa-Pro)	GRC 45 (bB2)	TUR19 (bB2)	RGBMC**S$_R$**S$_O$	2.21×10^{-17}	6.18
	8963 (CP)	TUR242 (bB2)	TUR97 (bB2)	RGBMC**S$_R$**S$_O$	1.45×10^{-19}	5.68
GRC 53	671 (P1)	TUR86 (bB2)	RN98 (wB1)	**R**GBMCS$_R$S$_O$	2.17×10^{-32}	6.37
	6041 (VPg)	IS1 (bB2)	GRC69 (bB2)	RGBMC**S$_R$**S$_O$	5.28×10^{-50}	4.97
GRC 55	7120 (NIa-Pro)	GRC 45 (bB2)	TUR19 (bB2)	RGBMC**S$_R$**S$_O$	8.03×10^{-17}	5.21
	8963 (CP)	TUR242 (bB2)	TUR97 (bB2)	RG**B**MCS$_R$S$_O$	9.08×10^{-20}	4.41
GRC 58	671 (P1)	TUR86 (bB2)	RN98 (wB1)	**R**GBMCS$_R$S$_O$	2.17×10^{-32}	5.21
	6041 (VPg)	IS1 (bB2)	GRC 69 (bB2)	RGBMC**S$_R$**S$_O$	4.85×10^{-50}	6.10
GRC 59	7120 (NIa-Pro)	GRC 45 (bB2)	TUR19 (bB2)	RBMC**S$_R$**S$_O$	4.94×10^{-11}	3.95
	8963 (CP)	TUR242 (bB2)	TUR97 (bB2)	RGBMC**S$_R$**S$_O$	9.70×10^{-19}	4.05
GRC 60	260 (P1)	GRC 65 (bB2)	TUR19 (bB2)	RGBMC**S$_R$**	1.85×10^{-24}	2.26
	2523 (HC-Pro) – 3327 (P3)	GRC 51 (bB2)	GRC 69 (bB2)	**R**GBMCS$_R$S$_O$	4.77×10^{-17}	4.41, 3.86
	7120 (NIa-Pro)	GRC 45 (bB2)	TUR19 (bB2)	RBMC**S$_R$**S$_O$	1.98×10^{-17}	3.86
	8963 (CP)	TUR242 (bB2)	TUR97 (bB2)	RGBMC**S$_R$**S$_O$	2.03×10^{-18}	3.97
GRC 61	260 (P1)	GRC 65 (bB2)	TUR19 (bB2)	RGBMC**S$_R$**	1.85×10^{-24}	2.26
	2523 (HC-Pro) – 3327 (P3)	GRC 51 (bB2)	GRC 69 (bB2)	**R**GBMCS$_R$S$_O$	5.23×10^{-17}	4.41, 3.86
	7120 (NIa-Pro)	GRC 45 (bB2)	TUR19 (bB2)	RBMC**S$_R$**S$_O$	3.71×10^{-17}	4.03
	8963 (CP)	TUR242 (bB2)	TUR97 (bB2)	RGBMC**S$_R$**S$_O$	2.03×10^{-18}	4.01
GRC 62	1842 (HC-Pro)	TUR55 (bB2)	TUR244 (bB2)	RGBMC**S$_R$**S$_O$	1.96×10^{-34}	6.35
	7120 (NIa-Pro)	GRC 45 (bB2)	TUR19 (bB2)	RGBMC**S$_R$**S$_O$	2.41×10^{-12}	6.18
	8963 (CP)	TUR242 (bB2)	TUR97 (bB2)	RGBMC**S$_R$**S$_O$	2.18×10^{-18}	4.48
GRC 63	260 (P1)	GRC 65 (bB2)	TUR19 (bB2)	RGBMC**S$_R$**	1.85×10^{-24}	2.26
	2523 (HC-Pro) – 3327 (P3)	GRC 51 (bB2)	GRC 69 (bB2)	**R**GBMCS$_R$S$_O$	2.14×10^{-16}	4.03
	7120 (NIa-Pro)	GRC 45 (bB2)	TUR19 (bB2)	BMC**S$_R$**S$_O$	3.93×10^{-17}	4.13
	8963 (CP)	TUR242 (bB2)	TUR97 (bB2)	RGBMC**S$_R$**S$_O$	2.03×10^{-18}	6.02
GRC 65	1842 (HC-Pro)	TUR55 (bB2)	TUR244 (bB2)	**R**GBMCS$_R$S$_O$	2.65×10^{-36}	4.13
	7120 (NIa-Pro)	GRC 45 (bB2)	TUR19 (bB2)	RGBMC**S$_R$**S$_O$	5.38×10^{-26}	5.13
	8963 (CP)	TUR242 (bB2)	TUR97 (bB2)	RGBMC**S$_R$**S$_O$	7.22×10^{-17}	4.63
GRC 69	7780 (NIb)	TUR242 (bB2)	TUR51 (bB2)	**R**GBMCS$_R$S$_O$	3.00×10^{-45}	6.56
	8963 (CP)	TUR242 (bB2)	TUR97 (bB2)	RGBMC**S$_R$**S$_O$	9.79×10^{-52}	7.23
GRC 70	7120 (NIa-Pro)	GRC 45 (bB2)	TUR19 (bB2)	RBMC**S$_R$**S$_O$	7.95×10^{-14}	6.89
	8963 (CP)	TUR242 (bB2)	TUR97 (bB2)	RMC**S$_R$**S$_O$	6.04×10^{-18}	4.03
GRC 71	5005 (CI)	TUR57 (bB2)	GRC 69 (bB2)	RG**B**MCS$_R$S$_O$	1.21×10^{-46}	6.69
	5005 (CI) – 7780 (NIb)	TUR242 (bB2)	TUR51 (bB2)	**R**GBMCS$_R$S$_O$	3.44×10^{-37}	7.02
	8963 (CP)	TUR242 (bB2)	TUR97 (bB2)	RGBMC**S$_R$**S$_O$	2.82×10^{-18}	5.68
Iran						
IRN BRE2	845 (P1)	CHL13 (ABR)	NZ412 (wB3)	RGBM**S$_R$**S$_O$	5.22×10^{-17}	3.20
	1632 (HC-Pro)	MYD015J (ABR)	AT181J (ABR)	**R**GBMCS$_R$S$_O$	4.95×10^{-95}	6.54
	9448 (CP)	59J (ABR)	TUR13 (ABR)	**R**GBCS$_R$S$_O$	3.12×10^{-13}	3.56
IRN BRE7	845 (P1)	CHL13 (ABR)	NZ412 (wB3)	RGBM**S$_R$**S$_O$	6.91×10^{-18}	3.20
	1632 (HC-Pro)	MYD015J (ABR)	AT181J (ABR)	**R**GBMCS$_R$S$_O$	1.59×10^{-91}	6.54
	9448 (CP)	59J (ABR)	TUR13 (ABR)	**R**GBS$_R$S$_O$	4.66×10^{-12}	3.56
IRN BRE12	845 (P1)	CHL13 (ABR)	NZ412 (wB3)	RGBM**S$_R$**S$_O$	5.04×10^{-18}	3.20
	1632 (HC-Pro)	MYD015J (ABR)	AT181J (ABR)	**R**GBMCS$_R$S$_O$	6.35×10^{-96}	6.54
	9448 (CP)	59J (ABR)	TUR13 (ABR)	**R**GBCS$_R$S$_O$	3.61×10^{-13}	3.56
IRN BRE19	8133 (NIb)	KWB779J (bBR)	NID119J (bBR)	RG**B**MCS$_R$S$_O$	2.68×10^{-45}	7.02
IRN BRSh12	744 (P1)	CHL13 (ABR)	NZ412 (wB3)	RBC**S$_R$**S$_O$	9.06×10^{-14}	3.42
	1632 (HC-Pro)	MYD015J (ABR)	AT181J (ABR)	**R**GBMCS$_R$S$_O$	7.59×10^{-106}	6.54
	9448 (CP)	59J (ABR)	TUR13 (ABR)	**R**GBCS$_R$S$_O$	2.79×10^{-13}	3.56
IRN CM1	1455 (HC-Pro)	IRN MB6 (Ir2)	IRN M14 (Ir2)	RG**B**MCS$_R$S$_O$	2.67×10^{-26}	3.67
IRN CnQ	5608 (CI)	IRN MM1 (Ir2)	IRN ST (Ir2)	RG**B**MCS$_R$S$_O$	3.44×10^{-15}	3.78
IRN EB1	9180 (CP)	IRN CV2 (bB2)	IRN TRa6 (bB2)	RGBMC**S$_R$**S$_O$	5.59×10^{-12}	4.37
IRN ER1	9180 (CP)	IRN CV2 (bB2)	IRN TRa6 (bB2)	**R**GBMCS$_R$S$_O$	2.86×10^{-8}	4.37
IRN ES1	9180 (CP)	IRN CV2 (bB2)	IRN TRa6 (bB2)	RGBMC**S$_R$**S$_O$	4.02×10^{-12}	4.37
IRN EM1	1455 (HC-Pro)	IRN MB6 (Ir2)	IRN M14 (Ir2)	RG**B**MCS$_R$S$_O$	3.15×10^{-26}	4.01
IRN M14	641 (P1) – 3907 (CI)	IRN MY57 (Ir2)	IRN MEr5 (Ir2)	RGBMC**S$_R$**S$_O$	4.25×10^{-21}	5.65
IRN M15	641 (P1) – 3907 (CI)	IRN MY57 (Ir2)	IRN MEr5 (Ir2)	RGBMC**S$_R$**S$_O$	2.21×10^{-21}	5.65

[a]Recombination sites detected in the turnip mosaic virus genomes by the recombination detection programs, from the aligned sequences of the likely recombinant and its 'parental isolates'. The nucleotide position shows locations of individual genes numbered as in UK 1 genome[20]. P1; protein 1, HC-Pro; helper component proteinase protein, P3; protein 3, CI; cylindrical inclusion protein, VPg; genome linked viral protein, NIb; nuclear inclusion b protein and CP; coat protein

[b]bB1; basal-B1 subgroup, bB2; basal-B2 subgroup, Ir1; Iranian 1 subgroup, Ir2; Iranian 2 subgroup, bBR; basal-BR group, ABR; Asian-BR group, wB1; world-B1 subgroup, wB2; world-B2 subgroup, wB3; world-B3 subgroup.

[c]Recombinant isolates identified by the recombination detection programs: R (RDP), G (GENECONV), B (BOOTSCAN), M (MAXCHI), C (CHIMAERA) and S$_R$ (SISCAN) programs in RDP4 package[21], and S$_O$ (SISCAN total nucleotide site analysis) in original SISCAN version 2[22] program. The analyses were done using default settings and a Bonferroni-corrected *P*-value cut-off of 0.01 in RDP4.

[d]The reported *P*-value is for the program in bold type and underlined in RDP4 package and is the smallest *P*-value among the isolates calculated for the region in question.

[e]Both of the parental isolates of the recombination sites had *Z*-values greater than 3 in total nucleotide site analysis in S$_O$ of the SISCAN version 2 program; thus lower *Z*-value of one of the parents identified are shown.

Supplementary Table S2. Continued.

Isolate	Nucleotide position and protein encoding region	Parental isolate and subgroup		Recombination detection program	*P*-value	*Z*-value
		Major	Minor			
IRN MEr1	6912 (NIa-Pro)	IRN MB6 (Ir2)	IRN DM (Ir2)	**R**GBMCS$_R$S$_O$	4.92×10^{-55}	6.82
IRN MEr3	6912 (NIa-Pro)	IRN MB6 (Ir2)	IRN DM (Ir2)	**R**GBMCS$_R$S$_O$	1.53×10^{-55}	6.82
IRN MEr5	6912 (NIa-Pro)	IRN MB6 (Ir2)	IRN DM (Ir2)	**R**GBMCS$_R$S$_O$	1.98×10^{-56}	6.82
IRN MM1	1455 (HC-Pro)	IRN MB6 (Ir2)	IRN M14 (Ir2)	RG**B**MCS$_R$S$_O$	2.72×10^{-23}	4.89
IRN REY4	5608 (CI)	IRN MM1 (Ir2)	IRN DM (Ir2)	RG**B**MCS$_R$S$_O$	4.46×10^{-37}	5.69
IRN RGhE	1455 (HC-Pro)	IRN MB6 (Ir2)	IRN M14 (Ir2)	RG**B**MCS$_R$S$_O$	1.01×10^{-27}	3.67
IRN RK	1455 (HC-Pro)	IRN MM1 (Ir2)	IRN ST (Ir2)	RB**M**S$_R$S$_O$	7.59×10^{-12}	4.33
IRN Rkaraj	1455 (HC-Pro)	IRN M14 (Ir2)	IRN ST (Ir2)	B**M**C	2.28×10^{-10}	2.21
IRN RRSh30	744 (P1)	CHL13 (ABR)	NZ412 (wB3)	RBC**S$_R$**S$_O$	9.06×10^{-14}	3.42
	1632 (HC-Pro)	MYD015J (ABR)	AT181J (ABR)	**R**GBMCS$_R$S$_O$	7.59×10^{-106}	6.54
	9448 (CP)	59J (ABR)	TUR13 (ABR)	RBM**S$_R$**S$_O$	1.52×10^{-13}	3.56
IRN RS2	744 (P1)	CHL13 (ABR)	NZ412 (wB3)	RB**C**S$_O$	5.18×10^{-10}	3.22
	1632 (HC-Pro)	MYD015J (ABR)	AT181J (ABR)	**R**GBMCS$_R$S$_O$	1.73×10^{-107}	6.54
	9448 (CP)	59J (ABR)	TUR13 (ABR)	RG**B**CS$_R$S$_O$	1.78×10^{-14}	3.56
IRN RS3	744 (P1)	CHL13 (ABR)	NZ412 (wB3)	RB**C**S$_O$	5.18×10^{-10}	3.22
	1632 (HC-Pro)	MYD015J (ABR)	AT181J (ABR)	**R**GBMCS$_R$S$_O$	1.73×10^{-107}	6.54
	9448 (CP)	59J (ABR)	TUR13 (ABR)	RG**B**CS$_R$S$_O$	1.78×10^{-14}	3.56
IRN ShA	9180 (CP)	IRN CV2 (bB2)	IRN TRa6 (bB2)	RGBMC**S$_R$**S$_O$	4.43×10^{-12}	4.02
IRN SRSh37	744 (P1)	CHL13 (ABR)	NZ412 (wB3)	RB**C**S$_O$	3.15×10^{-10}	3.22
	1632 (HC-Pro)	MYD015J (ABR)	AT181J (ABR)	**R**GBMCS$_R$S$_O$	4.45×10^{-107}	6.54
	9448 (CP)	59J (ABR)	TUR13 (ABR)	RG**B**CS$_R$S$_O$	1.22×10^{-15}	3.56
IRN SS5	9180 (CP)	IRN CV2 (bB2)	IRN TRa6 (bB2)	RGBMC**S$_R$**S$_O$	4.08×10^{-12}	3.99
IRN TKE	1455 (HC-Pro)	IRN MM1 (Ir2)	IRN ST (Ir2)	RB**M**S$_R$S$_O$	2.39×10^{-14}	4.33
IRN TH	1455 (HC-Pro)	IRN MM1 (Ir2)	IRN ST (Ir2)	RG**B**MCS$_R$S$_O$	1.00×10^{-14}	4.33
IRN TM5	1455 (HC-Pro)	IRN MB6 (Ir2)	IRN M14 (Ir2)	RG**B**MCS$_R$S$_O$	2.19×10^{-27}	3.67
IRN TSh8	1151 (P1)	IRN RaNi3 (Ir1)	IRN Tlm1 (Ir1)	RB**S$_R$**S$_O$	1.27×10^{-13}	3.60
IRN TOFS6	1455 (HC-Pro)	IRN MM1 (Ir2)	IRN ST (Ir2)	RBM**C**S$_R$S$_O$	5.03×10^{-13}	4.33
IRN TuK33	1455 (HC-Pro)	IRN ST (Ir2)	IRN EM1 (Ir2)	**R**GBMCS$_R$S$_O$	3.51×10^{-22}	4.68
IRN TuM5	1455 (HC-Pro)	IRN MB6 (Ir2)	IRN M14 (Ir2)	RG**B**MCS$_R$S$_O$	2.86×10^{-24}	3.67
IRN WRN8	744 (P1)	CHL13 (ABR)	NZ412 (wB3)	RBMC**S$_R$**S$_O$	1.41×10^{-13}	3.33
	1632 (HC-Pro)	MYD015J (ABR)	AT181J (ABR)	**R**GBMCS$_R$S$_O$	4.34×10^{-97}	6.54
	9448 (CP)	59J (ABR)	TUR13 (ABR)	**R**GBS$_R$S$_O$	5.61×10^{-13}	3.56
IRN WRN9	744 (P1)	CHL13 (ABR)	NZ412 (wB3)	RBMC**S$_R$**S$_O$	1.41×10^{-13}	3.33
	1632 (HC-Pro)	MYD015J (ABR)	AT181J (ABR)	RG**B**MCS$_R$S$_O$	8.71×10^{-98}	6.66
	9448 (CP)	59J (ABR)	TUR13 (ABR)	**R**GBS$_R$S$_O$	5.10×10^{-13}	3.40
IRN WRN10	744 (P1)	CHL13 (ABR)	NZ412 (wB3)	RBMC**S$_R$**S$_O$	1.41×10^{-13}	3.33
	1632 (HC-Pro)	MYD015J (ABR)	AT181J (ABR)	RG**B**MCS$_R$S$_O$	1.22×10^{-97}	6.66
	9448 (CP)	59J (ABR)	TUR13 (ABR)	**R**GBS$_R$S$_O$	4.13×10^{-12}	3.40
IRN WRN11	744 (P1)	CHL13 (ABR)	NZ412 (wB3)	RBMC**S$_R$**S$_O$	1.41×10^{-13}	3.33
	1632 (HC-Pro)	MYD015J (ABR)	AT181J (ABR)	RG**B**MCS$_R$S$_O$	4.28×10^{-98}	6.66
	9448 (CP)	59J (ABR)	TUR13 (ABR)	**R**GBS$_R$S$_O$	3.94×10^{-12}	3.40
IRN WRSh10	744 (P1)	CHL13 (ABR)	NZ412 (wB3)	RBMC**S$_R$**S$_O$	1.41×10^{-13}	3.33
	1632 (HC-Pro)	MYD015J (ABR)	AT181J (ABR)	**R**GBMCS$_R$S$_O$	3.87×10^{-100}	6.66
	9448 (CP)	59J (ABR)	TUR13 (ABR)	**R**GBS$_R$S$_O$	4.60×10^{-12}	3.40
IRN WRSh12	744 (P1)	CHL13 (ABR)	NZ412 (wB3)	RBMC**S$_R$**S$_O$	1.41×10^{-13}	3.33
	1632 (HC-Pro)	MYD015J (ABR)	AT181J (ABR)	**R**GBMCS$_R$S$_O$	3.18×10^{-107}	6.66
	9448 (CP)	59J (ABR)	TUR13 (ABR)	**R**GBS$_R$S$_O$	4.13×10^{-12}	3.40
IRN WRSh20	744 (P1)	CHL13 (ABR)	NZ412 (wB3)	RBMC**S$_R$**S$_O$	1.41×10^{-13}	3.33
	1632 (HC-Pro)	MYD015J (ABR)	AT181J (ABR)	**R**GBMCS$_R$S$_O$	1.72×10^{-107}	6.66
	9448 (CP)	59J (ABR)	TUR13 (ABR)	**R**GBS$_R$S$_O$	4.13×10^{-12}	3.40
IRN WRSh24	744 (P1)	CHL13 (ABR)	NZ412 (wB3)	RBMC**S$_R$**S$_O$	1.41×10^{-13}	3.33
	1632 (HC-Pro)	MYD015J (ABR)	AT181J (ABR)	**R**GBMCS$_R$S$_O$	1.70×10^{-105}	6.66
	9448 (CP)	59J (ABR)	TUR13 (ABR)	**R**GBS$_R$S$_O$	3.57×10^{-13}	3.40
IRN WRSh32	744 (P1)	CHL13 (ABR)	NZ412 (wB3)	RBMC**S$_R$**S$_O$	1.41×10^{-13}	3.33
	1632 (HC-Pro)	MYD015J (ABR)	AT181J (ABR)	**R**GBMCS$_R$S$_O$	1.70×10^{-105}	6.66
	9448 (CP)	59J (ABR)	TUR13 (ABR)	**R**GBS$_R$S$_O$	3.57×10^{-13}	3.40
IRN WRSh42	744 (P1)	CHL13 (ABR)	NZ412 (wB3)	RBMC**S$_R$**S$_O$	1.41×10^{-13}	3.33
	1632 (HC-Pro)	MYD015J (ABR)	AT181J (ABR)	**R**GBMCS$_R$S$_O$	1.55×10^{-107}	6.66
	9448 (CP)	59J (ABR)	TUR13 (ABR)	**R**GBS$_R$S$_O$	2.85×10^{-13}	3.40
IRN WRSh203	744 (P1)	CHL13 (ABR)	NZ412 (wB3)	RBMC**S$_R$**S$_O$	1.41×10^{-13}	3.33
	1632 (HC-Pro)	MYD015J (ABR)	AT181J (ABR)	**R**GBMCS$_R$S$_O$	1.39×10^{-105}	6.66
	9448 (CP)	59J (ABR)	TUR13 (ABR)	**R**GBS$_R$S$_O$	1.94×10^{-13}	3.40
IRN WRWA5	744 (P1)	CHL13 (ABR)	NZ412 (wB3)	RBMC**S$_R$**S$_O$	1.41×10^{-13}	3.33
	1632 (HC-Pro)	MYD015J (ABR)	AT181J (ABR)	RG**B**MCS$_R$S$_O$	8.27×10^{-100}	6.66
	9448 (CP)	59J (ABR)	TUR13 (ABR)	**R**GBS$_R$S$_O$	3.35×10^{-12}	3.40
IRN WRWA14	744 (P1)	CHL13 (ABR)	NZ412 (wB3)	RBMC**S$_R$**S$_O$	1.41×10^{-13}	3.33
	1632 (HC-Pro)	MYD015J (ABR)	AT181J (ABR)	RG**B**MCS$_R$S$_O$	4.91×10^{-100}	6.66
	9448 (CP)	59J (ABR)	TUR13 (ABR)	**R**GBS$_R$S$_O$	4.13×10^{-12}	3.40
IRN ZE	845 (P1)	CHL13 (ABR)	NZ412 (wB3)	RGBM**S$_R$**S$_O$	4.31×10^{-12}	3.20
	1632 (HC-Pro)	MYD015J (ABR)	AT181J (ABR)	**R**GBMCS$_R$S$_O$	1.42×10^{-97}	6.54
	9448 (CP)	59J (ABR)	TUR13 (ABR)	**R**GBCS$_R$S$_O$	3.57×10^{-13}	3.56
Turkey						
TUR1	1176 (P1)	TUR73 (wB2)	CAR37 (wB1)	RGBMC**S$_R$**S$_O$	4.72×10^{-54}	4.65
	2508 (HC-Pro) – 6277 (VPg)	DEU 5 (wB2)	GRC 31 (wB3)	R**G**BMCS$_R$S$_O$	1.05×10^{-60}	5.06, 1.56
TUR3	1221 (HC-Pro)	TUR9 (ABR)	DEU 1 (wB2)	RGB**S$_R$**S$_O$	1.05×10^{-14}	2.61
	2508 (HC-Pro) – 6277 (VPg)	QCa (wB2)	GRC 31 (wB3)	RG**B**MCS$_R$S$_O$	3.96×10^{-65}	4.87
TUR4	1221 (HC-Pro)	TUR9 (ABR)	DEU 1 (wB2)	RGB**S$_R$**S$_O$	1.05×10^{-14}	2.61
	2508 (HC-Pro) – 6277 (VPg)	QCa (wB2)	GRC 31 (wB3)	RG**B**MCS$_R$S$_O$	3.96×10^{-65}	4.87
TUR5	1221 (HC-Pro)	TUR9 (ABR)	DEU 1 (wB2)	RGB**S$_R$**S$_O$	1.05×10^{-14}	2.61
	2508 (HC-Pro) – 6277 (VPg)	QCa (wB2)	GRC 31 (wB3)	RG**B**MCS$_R$S$_O$	3.96×10^{-65}	4.87
TUR6	1221 (HC-Pro)	TUR9 (ABR)	DEU 1 (wB2)	RGB**S$_R$**S$_O$	1.05×10^{-14}	2.61
	2508 (HC-Pro) – 6277 (VPg)	QCa (wB2)	GRC 31 (wB3)	RG**B**MCS$_R$S$_O$	3.96×10^{-65}	4.87
TUR7	1221 (HC-Pro)	TUR9 (ABR)	DEU 1 (wB2)	RGB**S$_R$**S$_O$	1.05×10^{-14}	2.61
	2508 (HC-Pro) – 6277 (VPg)	QCa (wB2)	GRC 31 (wB3)	RG**B**MCS$_R$S$_O$	3.96×10^{-65}	4.87
TUR8	1221 (HC-Pro)	TUR9 (ABR)	DEU 1 (wB2)	RGB**S$_R$**S$_O$	1.05×10^{-14}	2.61
	2508 (HC-Pro) – 6277 (VPg)	QCa (wB2)	GRC 31 (wB3)	RG**B**MCS$_R$S$_O$	3.96×10^{-65}	4.87
TUR13	610 (P1) – 1540 (P1)	TUR9 (ABR)	TUR52 (ABR)	RGBMC**S$_R$**S$_O$	2.07×10^{-33}	7.96
	5973 (VPg) – 8813 (CP)	TUR10 (ABR)	TUR52 (ABR)	RGBMC**S$_R$**S$_O$	4.74×10^{-34}	5.62
TUR14	610 (P1) – 1540 (P1)	TUR9 (ABR)	TUR52 (ABR)	RGBMC**S$_R$**S$_O$	5.17×10^{-35}	7.96
	5973 (VPg) – 8813 (CP)	TUR10 (ABR)	TUR52 (ABR)	RGBMC**S$_R$**S$_O$	3.95×10^{-34}	5.62
TUR16	7120 (NIa-Pro)	GRC 45 (bB2)	TUR19 (bB2)	RGBMC**S$_R$**S$_O$	1.97×10^{-12}	4.33
	8112 (NIb)	GRC 69 (bB2)	TUR36 (bB2)	RG**B**MCS$_R$S$_O$	3.54×10^{-36}	5.16
TUR19	1320 (HC-Pro)	GRC 44 (bB2)	TUR95 (bB2)	**R**GBMCS$_R$S$_O$	6.74×10^{-24}	3.10
	1320 (HC-Pro) – 2543 (HC-Pro)	TUR95 (bB2)	GRC 44 (bB2)	RGBMC**S$_R$**S$_O$	4.45×10^{-26}	3.47, 3.67
	7654 (NIb) – 8460 (NIb)	TUR242 (bB2)	TUR86 (bB2)	**R**GBMCS$_R$S$_O$	1.84×10^{-21}	3.17, 4.09
TUR25	7120 (NIa-Pro)	GRC 45 (bB2)	TUR19 (bB2)	RBMC**S$_R$**S$_O$	3.45×10^{-17}	4.33
	8112 (NIb)	GRC 69 (bB2)	TUR36 (bB2)	RG**B**MCS$_R$S$_O$	8.63×10^{-42}	5.16
TUR30	1710 (HC-Pro)	A64 (bB1)	TUR56 (bB2)	**R**GBMCS$_R$S$_O$	1.15×10^{-117}	6.86
TUR31	1710 (HC-Pro)	A64 (bB1)	TUR56 (bB2)	**R**GBMCS$_R$S$_O$	8.52×10^{-119}	6.86
TUR34	260 (P1)	TUR19 (bB2)	GRC 44 (bB2)	RGBMC**S$_R$**S$_O$	6.03×10^{-33}	3.17
	7120 (NIa-Pro)	GRC 45 (bB2)	TUR19 (bB2)	**R**BMCS$_R$S$_O$	2.47×10^{-19}	4.05
	9130 (CP)	TUR57 (bB2)	TUR27 (ABR)	RG**B**	2.09×10^{-19}	2.64

Supplementary Table S2. Continued.

Isolate	Nucleotide position and protein encoding region	Parental isolate and subgroup		Recombination detection program	*P*-value	*Z*-value
		Major	Minor			
TUR36	7120 (NIa-Pro)	GRC 45 (bB2)	TUR19 (bB2)	RGBMC**S$_R$**S$_O$	5.52×10^{-18}	3.74
	8638 (NIb)	TUR57 (bB2)	Eru 1D (bB1)	RG**B**MCS$_R$S$_O$	8.03×10^{-78}	6.99
TUR40	260 (P1)	TUR19 (bB2)	GRC 44 (bB2)	RGBMC**S$_R$**S$_O$	6.03×10^{-33}	3.17
	7120 (NIa-Pro)	GRC 45 (bB2)	TUR19 (bB2)	**R**BMCS$_R$S$_O$	2.47×10^{-19}	4.05
	9130 (CP)	TUR57 (bB2)	TUR27 (ABR)	RG**B**	2.09×10^{-19}	2.64
TUR41	260 (P1)	TUR19 (bB2)	GRC 44 (bB2)	RGBMC**S$_R$**S$_O$	6.03×10^{-33}	3.17
	7120 (NIa-Pro)	GRC 45 (bB2)	TUR19 (bB2)	**R**BMCS$_R$S$_O$	2.47×10^{-19}	4.05
	9130 (CP)	TUR57 (bB2)	TUR27 (ABR)	RG**B**	2.09×10^{-19}	2.64
TUR42	260 (P1)	TUR19 (bB2)	GRC 44 (bB2)	RGBMC**S$_R$**S$_O$	6.03×10^{-33}	3.17
	7120 (NIa-Pro)	GRC 45 (bB2)	TUR19 (bB2)	**R**BMCS$_R$S$_O$	2.47×10^{-19}	4.05
	9130 (CP)	TUR57 (bB2)	TUR27 (ABR)	RG**B**	2.09×10^{-19}	2.64
TUR43	260 (P1)	TUR19 (bB2)	GRC 44 (bB2)	RGBMC**S$_R$**S$_O$	6.03×10^{-33}	3.17
	7120 (NIa-Pro)	GRC 45 (bB2)	TUR19 (bB2)	**R**BMCS$_R$S$_O$	2.47×10^{-19}	4.05
	9130 (CP)	TUR57 (bB2)	TUR27 (ABR)	RG**B**	2.09×10^{-19}	2.64
TUR44	260 (P1)	TUR19 (bB2)	GRC 44 (bB2)	RGBMC**S$_R$**S$_O$	6.03×10^{-33}	3.17
	7120 (NIa-Pro)	GRC 45 (bB2)	TUR19 (bB2)	**R**BMCS$_R$S$_O$	2.47×10^{-19}	4.05
	9130 (CP)	TUR57 (bB2)	TUR27 (ABR)	RG**B**	2.09×10^{-19}	2.64
TUR45	260 (P1)	TUR19 (bB2)	GRC 44 (bB2)	RGBMC**S$_R$**S$_O$	6.03×10^{-33}	3.17
	7120 (NIa-Pro)	GRC 45 (bB2)	TUR19 (bB2)	**R**BMCS$_R$S$_O$	2.47×10^{-19}	4.05
	9130 (CP)	TUR57 (bB2)	TUR27 (ABR)	RG**B**	2.09×10^{-19}	2.64
TUR47	260 (P1)	TUR19 (bB2)	GRC 44 (bB2)	RGBMC**S$_R$**S$_O$	6.03×10^{-33}	3.17
	7120 (NIa-Pro)	GRC 45 (bB2)	TUR19 (bB2)	**R**BMCS$_R$S$_O$	2.47×10^{-19}	4.05
	9130 (CP)	TUR57 (bB2)	TUR27 (ABR)	RG**B**	2.09×10^{-19}	2.64
TUR49	706 (P1)	TUR86 (bB2)	TUR13 (ABR)	**R**GBS$_R$S$_O$	2.03×10^{-14}	3.08
TUR50	8963 (CP)	TUR55 (bB2)	IS1 (bB2)	RG**B**MCS$_R$S$_O$	1.50×10^{-34}	3.63
TUR51	7120 (NIa-Pro)	GRC 45 (bB2)	TUR19 (bB2)	RBMC**S$_R$**S$_O$	2.36×10^{-17}	4.33
	8112 (NIb)	GRC 69 (bB2)	TUR36 (bB2)	**R**GBMCS$_R$S$_O$	3.14×10^{-41}	5.16
TUR55	7120 (NIa-Pro)	GRC 45 (bB2)	TUR19 (bB2)	RBMC**S$_R$**S$_O$	5.65×10^{-18}	4.33
	8112 (NIb)	GRC 69 (bB2)	TUR36 (bB2)	R**G**BMCS$_R$S$_O$	1.11×10^{-38}	5.16
TUR56	6222 (VPg)	TUR242 (bB2)	IS1 (bB2)	**R**GBMCS$_R$S$_O$	1.16×10^{-18}	4.11
TUR57	7120 (NIa-Pro)	GRC 45 (bB2)	TUR19 (bB2)	RBMC**S$_R$**S$_O$	7.17×10^{-19}	4.33
	8112 (NIb)	GRC 69 (bB2)	TUR36 (bB2)	RG**B**MCS$_R$S$_O$	1.73×10^{-36}	5.16
TUR58	1221 (HC-Pro)	TUR9 (ABR)	DEU 1 (wB2)	RGB**S$_R$**S$_O$	1.05×10^{-14}	2.61
	2508 (HC-Pro) – 6277 (VPg)	QCa (wB2)	GRC 31 (wB3)	RG**B**MCS$_R$S$_O$	3.96×10^{-65}	4.87
TUR59	7120 (NIa-Pro)	GRC 45 (bB2)	TUR19 (bB2)	RGBMC**S$_R$**S$_O$	6.84×10^{-18}	3.21
	8112 (NIb)	GRC 69 (bB2)	TUR36 (bB2)	RGBMC**S$_R$**S$_O$	5.58×10^{-52}	6.03
	9180 (CP)	TUR57 (bB2)	TUR56 (bB2)	RG**B**MCS$_R$S$_O$	5.80×10^{-24}	4.36
TUR60	7120 (NIa-Pro)	GRC 45 (bB2)	TUR19 (bB2)	RGBMC**S$_R$**S$_O$	1.25×10^{-17}	3.21
	8112 (NIb)	GRC 69 (bB2)	TUR36 (bB2)	RBMC**S$_R$**S$_O$	1.84×10^{-51}	6.03
	9180 (CP)	TUR57 (bB2)	TUR56 (bB2)	RG**B**MCS$_R$S$_O$	5.80×10^{-24}	4.36
TUR61	7120 (NIa-Pro)	GRC 45 (bB2)	TUR19 (bB2)	RGBMC**S$_R$**S$_O$	1.08×10^{-17}	3.21
	8112 (NIb)	GRC 69 (bB2)	TUR36 (bB2)	RGBMC**S$_R$**S$_O$	1.00×10^{-51}	6.03
	9180 (CP)	TUR57 (bB2)	TUR56 (bB2)	RG**B**MCS$_R$S$_O$	1.86×10^{-24}	4.36
TUR62	1221 (HC-Pro)	TUR9 (ABR)	DEU 1 (wB2)	RGB**S$_R$**S$_O$	1.05×10^{-14}	2.61
	2508 (HC-Pro) – 6277 (VPg)	QCa (wB2)	GRC 31 (wB3)	RG**B**MCS$_R$S$_O$	3.96×10^{-65}	4.87
TUR63	1710 (HC-Pro)	TUR64 (bB1)	IRN TP1 (bB2)	RGBMC**S$_R$**S$_O$	3.51×10^{-35}	5.68
TUR64	2979 (P3)	Eru 1D (bB1)	GRC 43 (bB1)	RG**M**C	9.61×10^{-15}	2.26
	5532 (CI)	A64 (bB1)	Al (bB1)	RBMC**S$_R$**S$_O$	6.64×10^{-26}	4.38
TUR65	1221 (HC-Pro)	TUR9 (ABR)	DEU 1 (wB2)	RGB**S$_R$**S$_O$	1.05×10^{-14}	2.61
	2508 (HC-Pro) – 6277 (VPg)	QCa (wB2)	GRC 31 (wB3)	RG**B**MCS$_R$S$_O$	3.96×10^{-65}	4.87
TUR66	1221 (HC-Pro)	TUR9 (ABR)	DEU 1 (wB2)	RGB**S$_R$**S$_O$	1.05×10^{-14}	2.61
	2508 (HC-Pro) – 6277 (VPg)	QCa (wB2)	GRC 31 (wB3)	RG**B**MCS$_R$S$_O$	3.96×10^{-65}	4.87
TUR67	1221 (HC-Pro)	TUR9 (ABR)	DEU 1 (wB2)	RGB**S$_R$**S$_O$	1.05×10^{-14}	2.61
	2508 (HC-Pro) – 6277 (VPg)	QCa (wB2)	GRC 31 (wB3)	RG**B**MCS$_R$S$_O$	3.96×10^{-65}	4.87
TUR68	1221 (HC-Pro)	TUR9 (ABR)	DEU 1 (wB2)	RGB**S$_R$**S$_O$	1.05×10^{-14}	2.61
	2508 (HC-Pro) – 6277 (VPg)	QCa (wB2)	GRC 31 (wB3)	RG**B**MCS$_R$S$_O$	3.96×10^{-65}	4.87
TUR73	1221 (HC-Pro)	TUR9 (ABR)	DEU 1 (wB2)	RGB**S$_R$**S$_O$	1.05×10^{-14}	2.61
	2508 (HC-Pro) – 6277 (VPg)	QCa (wB2)	GRC 31 (wB3)	RG**B**MCS$_R$S$_O$	3.96×10^{-65}	4.87
TUR78	7120 (NIa-Pro)	GRC 45 (bB2)	TUR19 (bB2)	RBMC**S$_R$**S$_O$	6.72×10^{-18}	4.33
	8112 (NIb)	GRC 69 (bB2)	TUR36 (bB2)	**R**GBMCS$_R$S$_O$	1.48×10^{-38}	5.16
TUR80	1221 (HC-Pro)	TUR9 (ABR)	DEU 1 (wB2)	RGB**S$_R$**S$_O$	1.05×10^{-14}	2.61
	2508 (HC-Pro) – 6277 (VPg)	QCa (wB2)	GRC 31 (wB3)	RG**B**MCS$_R$S$_O$	3.96×10^{-65}	4.87
TUR84	1202 (P1) – 2371 (HC-Pro)	IRN S1 (bB2)	TUR56 (bB2)	**R**GBMCS$_R$S$_O$	9.01×10^{-25}	3.89, 4.01
	6222 (VPg)	GRC 58 (bB2)	TUR91 (bB2)	RG**B**MCS$_R$S$_O$	7.86×10^{-57}	5.21
TUR85	1202 (P1) – 2371 (HC-Pro)	IRN S1 (bB2)	TUR56 (bB2)	RG**B**MCS$_R$S$_O$	1.74×10^{-26}	3.89, 4.01
	6222 (VPg)	GRC 58 (bB2)	TUR91 (bB2)	RG**B**MCS$_R$S$_O$	2.25×10^{-57}	5.21
TUR86	1202 (P1) – 2371 (HC-Pro)	IRN S1 (bB2)	TUR56 (bB2)	**R**GBMCS$_R$S$_O$	3.69×10^{-25}	3.89, 4.01
TUR88	260 (P1)	TUR19 (bB2)	GRC 44 (bB2)	RGBMC**S$_R$**S$_O$	6.03×10^{-33}	3.17
	7120 (NIa-Pro)	GRC 45 (bB2)	TUR19 (bB2)	**R**BMCS$_R$S$_O$	2.47×10^{-19}	4.05
	9130 (CP)	TUR57 (bB2)	TUR27 (ABR)	RG**B**	2.09×10^{-19}	2.64
TUR90	260 (P1)	TUR19 (bB2)	GRC 44 (bB2)	RGBMC**S$_R$**S$_O$	6.03×10^{-33}	3.17
	7120 (NIa-Pro)	GRC 45 (bB2)	TUR19 (bB2)	**R**BMCS$_R$S$_O$	2.47×10^{-19}	4.05
	9130 (CP)	TUR57 (bB2)	TUR27 (ABR)	RG**B**	2.09×10^{-19}	2.64
TUR91	2386 (HC-Pro)	TUR79 (bB2)	TUR242 (bB2)	RGBMC**S$_R$**S$_O$	4.59×10^{-62}	6.03
	6222 (VPg)	TUR50 (bB2)	TUR85 (bB2)	RG**B**MCS$_R$S$_O$	2.66×10^{-63}	5.91
TUR95	260 (P1)	TUR19 (bB2)	GRC 44 (bB2)	RGBMC**S$_R$**S$_O$	6.03×10^{-33}	3.17
	7120 (NIa-Pro)	GRC 45 (bB2)	TUR19 (bB2)	**R**BMCS$_R$S$_O$	2.47×10^{-19}	4.05
	9130 (CP)	TUR57 (bB2)	TUR27 (ABR)	RG**B**	2.09×10^{-19}	2.64
TUR97	260 (P1)	TUR19 (bB2)	GRC 44 (bB2)	RGBMC**S$_R$**S$_O$	6.03×10^{-33}	3.17
	7120 (NIa-Pro)	GRC 45 (bB2)	TUR19 (bB2)	**R**BMCS$_R$S$_O$	2.47×10^{-19}	4.05
	9130 (CP)	TUR57 (bB2)	TUR27 (ABR)	RG**B**	2.09×10^{-19}	2.64
TUR106	1221 (HC-Pro)	TUR9 (ABR)	DEU 1 (wB2)	RGB**S$_R$**S$_O$	1.05×10^{-14}	2.61
	2508 (HC-Pro) – 6277 (VPg)	QCa (wB2)	GRC 31 (wB3)	RG**B**MCS$_R$S$_O$	3.96×10^{-65}	4.87

Supplementary Table S3. Estimates of dissemination pathways of turnip mosaic virus[a] in Asian Minor and its neighboring countries with European countries.

Group and Subgroup	Protein-coding region[b]															
	Major ORF (n=106)				HC-Pro* (n=329)				P3* (n=369)				NIb* (n=351)			
	From	To	YBP[c]	BF[d]	From	To	YBP	BF	From	To	YBP	BF	From	To	YBP	BF
basal-B (basal-B1+2)	Turkey (Adana)	Iran (Tehran)	162 (97-381)[e]	288	Turkey (Canakkale)	Greece (Thessaloniki)	123 (40-189)	149	Turkey (Adana)	Turkey (Urfa)	28 (15-37)	179	Turkey (Izmir)	Turkey (Aydin)	74 (25-102)	215
	Turkey (Bolu)	Turkey (Adana)	293 (125-421)	223	Turkey (Aydin)	Turkey (Konya)	84 (31-125)	121	Iran (Semnan)	Iran (Tehran)	51 (28-101)	151	Turkey (Canakkale)	Turkey (Izmir)	79 (26-108)	197
	Turkey (Adana)	Iran (Semnan)	151 (96-376)	159	Iran (Shiraz)	Iran (Kerman)	33 (13-55)	87	Italy (Campania)	Greece (Thessaloniki)	37 (20-79)	105	Iran (Semnan)	Iran (Tehran)	49 (15-97)	158
	Germany (Gatersleben)	Turkey (Bolu)	298 (121-429)	101	Iran (Semnan)	Iran (Tehran)	68 (24-125)	95	Turkey (Aydin)	Turkey (Konya)	61 (21-109)	103	Turkey (Aydin)	Turkey (Konya)	65 (25-106)	109
	Italy (Campania)	Greece (Thessaloniki)	91 (49-146)	22	Turkey (Izmir)	Turkey (Adana)	76 (30-120)	41	Iran (Tehran)	Iran (Shiraz)	35 (17-56)	98	Turkey (Adana)	Italy (Campania)	58 (32-120)	85
	Italy (Campania)	Italy (Piedmont)	212 (92-376)	23	Iran (Tehran)	Iran (Shiraz)	38 (19-72)	39	Turkey (Canakkale)	Turkey (Aydin)	81 (28-116)	58	Turkey (Konya)	Turkey (Mersin)	28 (15-42)	81
	Germany (Gatersleben)	Italy (Campania)	331 (157-497)	19	Greece (Thessaloniki)	Greece (Euboia)	88 (35-132)	28	Greece (Thessaloniki)	Greece (Euboia)	62 (25-112)	40	Turkey (Canakkale)	Greece (Thessaloniki)	95 (34-154)	58
					Turkey (Canakkale)	Turkey (Aydin)	102 (40-136)	22	Turkey (Adana)	Italy (Campania)	56 (27-96)	38	Greece (Patras)	Greece (Euboia)	52 (21-97)	39
					Turkey (Adana)	Iran (Semnan)	74 (31-126)	19	Turkey (Adana)	Iran (Semnan)	70 (29-122)	16	Greece (Thessaloniki)	Greece (Patras)	48 (21-105)	28
					Italy (Campania)	Greece (Thessaloniki)	122 (45-190)	15	Italy (Toscana)	Spain (Unknown)	54 (21-110)	97	Iran (Tehran)	Iran (Esfahan)	43 (26-75)	26
					Germany (Frankfurt)	Denmark (Not known)	56 (20-108)	148	Germany (Frankfurt)	Denmark (Not known)	29 (13-46)	53	Iran (Tehran)	Iran (Shiraz)	22 (13-68)	18
					Germany (Frankfurt)	Germany (Braunschweig)	37 (14-57)	91					Germany (Gatersleben)	Italy (Campania)	245 (111-408)	94
					Italy (Campania)	Italy (Piedmont)	48 (23-69)	84					Germany (Frunkfurt)	Denmark (Not known)	38 (16-59)	88
													Italy (Campania)	Italy (Piedmont)	49 (21-67)	27
basal-B2	Turkey (Bolu)	Turkey (Adana)	291 (103-419)	268	Turkey (Aydin)	Turkey (Konya)	79 (29-115)	139	Turkey (Adana)	Turkey (Urfa)	26 (13-35)	211	Turkey (Izmir)	Turkey (Aydin)	75 (26-107)	198
	Turkey (Adana)	Iran (Tehran)	171 (103-401)	241	Turkey (Canakkale)	Greece (Thessaloniki)	124 (41-185)	114	Iran (Semnan)	Iran (Tehran)	53 (26-92)	168	Turkey (Canakkale)	Turkey (Izmir)	80 (27-110)	153
	Turkey (Adana)	Iran (Semnan)	156 (99-382)	176	Iran (Shiraz)	Iran (Kerman)	31 (12-53)	97	Turkey (Aydin)	Turkey (Konya)	63 (20-108)	142	Iran (Semnan)	Iran (Tehran)	52 (16-99)	141
					Iran (Semnan)	Iran (Tehran)	69 (22-120)	92	Turkey (Canakkale)	Greece (Thessaloniki)	106 (36-165)	74	Turkey (Aydin)	Turkey (Konya)	61 (21-104)	103
					Turkey (Izmir)	Turkey (Adana)	76 (30-120)	58	Italy (Campania)	Greece (Thessaloniki)	39 (19-77)	65	Turkey (Konya)	Turkey (Mersin)	29 (12-40)	87
					Iran (Tehran)	Iran (Shiraz)	39 (17-69)	55	Iran (Tehran)	Iran (Shiraz)	38 (15-53)	64	Turkey (Adana)	Italy (Campania)	66 (33-121)	79
					Greece (Thessaloniki)	Greece (Euboia)	82 (32-129)	49	Turkey (Canakkale)	Turkey (Aydin)	83 (29-114)	46	Turkey (Canakkale)	Greece (Thessaloniki)	98 (35-157)	64
					Turkey (Canakkale)	Turkey (Aydin)	94 (33-135)	47	Greece (Thessaloniki)	Greece (Euboia)	60 (20-106)	41	Greece (Patras)	Greece (Euboia)	56 (19-101)	49
					Turkey (Adana)	Iran (Semnan)	78 (32-125)	36	Turkey (Adana)	Italy (Campania)	52 (25-92)	36	Greece (Thessaloniki)	Greece (Patras)	63 (22-108)	35
					Greece (Thessaloniki)	Greece (Patras)	93 (35-134)	20	Turkey (Adana)	Iran (Semnan)	71 (26-111)	29	Iran (Tehran)	Iran (Esfahan)	40 (20-71)	27
									Greece (Thessalonki)	Greece (Patras)	71 (26-113)	29	Iran (Tehran)	Iran (Shiraz)	38 (19-69)	21
Iranian (Iranian 1+2)	Iran (Tehran)	Iran (Markazi)	82 (39-125)	314	Iran (Tehran)	Iran (Markazi)	34 (13-43)	323	Iran (Tehran)	Iran (Markazi)	34 (14-48)	285	Iran (Tehran)	Iran (Markazi)	33 (15-46)	246
	Iran (Markazi)	Iran (Tehran)	61 (23-101)	197	Iran (Tehran)	Iran (Qom)	74 (23-108)	284	Iran (Markazi)	Iran (Tehran)	28 (13-39)	216	Iran (Tehran)	Iran (Qom)	62 (18-107)	197
	Iran (Qom)	Iran (Kerman)	93 (43-147)	187	Iran (Markazi)	Iran (Tehran)	29 (12-39)	211	Iran (Tehran)	Iran (Qom)	54 (21-104)	194	Iran (Markazi)	Iran (Esfahan)	26 (14-39)	71
	Iran (Tehran)	Iran (Azarbayjan-e-Sharqi)	55 (21-83)	84	Iran (Markazi)	Iran (Esfahan)	30 (12-41)	153	Iran (Markazi)	Iran (Esfahan)	23 (12-36)	90	Iran (Tehran)	Iran (Azarbayjan-e-Sharqi)	44 (20-75)	41
	Iran (Tehran)	Iran (Qom)	122 (60-187)	69	Iran (Tehran)	Iran (Azarbayjan-e-Sharqi)	41 (16-65)	75	Iran (Tehran)	Iran (Azarbayjan-e-Sharqi)	35 (14-49)	55				
					Iran (Esfahan)	Iran (Kerman)	27 (11-37)	28								

[a]Disseminations in Greece, Iran and Turkey (in black letter), and European countries (in red letter) are only listed.
[b]Major ORF (major open reading frame), HC-Pro* (partial helper-component proteinase), P3* (partial protein 3), NIb* (partial nuclear inclusion b)
[c]Years before present
[d]Bayes factor
[e]The 95% credibility intervals (CI) for each age estimate is given in parentheses.

Table S3. Continued.

Group and Subgroup	Protein-coding region Major ORF (n=106) From	To	YBP	BF	HC-Pro* (n=329) From	To	YBP	BF	P3* (n=369) From	To	YBP	BF	NIb* (n=351) From	To	YBP	BF
Iranian 2	Iran (Tehran)	Iran (Markazi)	77 (37-124)	281	Iran (Tehran)	Iran (Markazi)	39 (15-46)	220	Iran (Tehran)	Iran (Markazi)	32 (12-48)	175	Iran (Tehran)	Iran (Markazi)	31 (14-45)	188
	Iran (Markazi)	Iran (Tehran)	65 (25-112)	148	Iran (Tehran)	Iran (Qom)	72 (26-111)	215	Iran (Markazi)	Iran (Tehran)	29 (14-40)	127	Iran (Tehran)	Iran (Qom)	60 (19-105)	174
	Iran (Tehran)	Iran (Azarbayjan-e-Sharqi)	70 (35-121)	127	Iran (Markazi)	Iran (Tehran)	34 (16-42)	127	Iran (Tehran)	Iran (Qom)	48 (22-97)	111	Iran (Markazi)	Iran (Esfahan)	24 (16-45)	64
	Iran (Tehran)	Iran (Qom)	119 (58-179)	107	Iran (Markazi)	Iran (Esfahan)	29 (15-47)	106	Iran (Markazi)	Iran (Esfahan)	21 (10-34)	76	Iran (Tehran)	Iran (Azarbayjan-e-Sharqi)	43 (23-78)	47
	Iran (Qom)	Iran (Kerman)	90 (41-141)	54	Iran (Tehran)	Iran (Azarbayjan-e-Sharqi)	44 (15-71)	71	Iran (Tehran)	Iran (Azarbayjan-e-Sharqi)	36 (19-45)	59				
					Iran (Esfahan)	Iran (Kerman)	23 (15-39)	27								
basal-BR	Italy (Campania)	Iran (Shiraz)	231 (109-402)	74												
	Italy (Liguria)	Italy (Campania)	92 (52-151)	99												
Asian-BR									Iran (Gilan)	Iran (Azarbayjan-e-Gharbi)	26 (12-38)	189	Iran (Gilan)	Iran (Azarbayjan-e-Gharbi)	18 (9-26)	97
									Iran (Gilan)	Iran (Shiraz)	19 (8-28)	116	Iran (Gilan)	Iran (Shiraz)	23 (11-36)	54
									Turkey (Canakkale)	Iran (Gilan)	48 (23-71)	47				
world-B (world-B1+ 2+3)	UK (Liandudno)	Greece (Lamia)	282 (123-513)	124	Turkey (Canakkale)	Turkey (Bursa)	29 (15-51)	228	Turkey (Canakkale)	Turkey (Nigde)	26 (13-40)	158	Turkey (Canakkale)	Turkey (Nigde)	15 (9-25)	187
	Greece (Lamia)	Greece (Kavala)	82 (41-139)	108	Turkey (Bursa)	Turkey (Konya)	17 (11-35)	175	Turkey (Nigde)	Turkey (Adana)	18 (10-28)	141	Turkey (Canakkale)	Turkey (Konya)	19 (11-30)	147
	Greece (Lamia)	Greece (Thessaloniki)	152 (74-296)	54	Greece (Xanthi)	Greece (Kavala)	46 (23-62)	97	Turkey (Konya)	Turkey (Canakkale)	25 (16-40)	87	Greece (Lamia)	Greece (Thessaloniki)	27 (16-51)	22
	Berguim (Wavre)	Russia (Unknown)	312 (134-548)	41	Turkey (Canakkale)	Turkey (Nigde)	23 (14-44)	57	UK (Dorset)	Turkey (Konya)	35 (19-55)	58	UK (Liandudno)	Denmark (Unknown)	58 (20-87)	69
	Russia (Unknown)	Poland (Unknown)	51 (16-94)	24	Greece (Xanthi)	Greece (Thessaloniki)	41 (21-62)	46	UK (Dorset)	Greece (Xanthi)	45 (24-59)	36	Berguim (Wavre)	Russia (Unknown)	125 (48-245)	21
	Russia (Unknown)	Czech Republic (Ceske Budejovice)	95 (46-159)	16	Greece (Kavala)	Greece (Lamia)	21 (11-32)	13	UK (Liandudno)	Germany (Wuenzburg)	49 (26-64)	45	Czech Republic (Prague)	Poland (Not known)	26 (12-40)	18
					UK (Liandudno)	Germany (Wuenzburg)	64 (29-97)	49	Czech Republic (Prague)	Poland (Not known)	31 (16-58)	24				
					Germany (Not known)	UK (Winspit)	56 (23-87)	38	Berguim (Wavre)	UK (Dorset)	112 (58-186)	19				
					Berguim (Wavre)	Russia (Unknown)	81 (39-140)	13								
world-B2					Turkey (Canakkale)	Turkey (Bursa)	27 (13-49)	210					Turkey (Canakkale)	Turkey (Nigde)	18 (8-25)	203
					Turkey (Bursa)	Turkey (Konya)	23 (12-37)	198					Turkey (Canakkale)	Turkey (Konya)	23 (12-31)	101
					Turkey (Canakkale)	Turkey (Nigde)	20 (16-40)	80								
world-B3	Greece (Lamia)	Greece (Kavala)	84 (42-142)	174	Greece (Xanthi)	Greece (Kavala)	41 (20-58)	104	Turkey (Canakkale)	Turkey (Nigde)	25 (12-38)	103	Greece (Lamia)	Greece (Thessaloniki)	29 (14-64)	34
	Greece (Lamia)	Greece (Thessaloniki)	149 (72-285)	69	Greece (Xanthi)	Greece (Thessaloniki)	46 (22-63)	50	Turkey (Nigde)	Turkey (Adana)	16 (9-27)	97				
					Greece (Kavala)	Greece (Lamia)	20 (11-31)	25	Turkey (Konya)	Turkey (Canakkale)	28 (13-41)	49				
									UK (Dorset)	Turkey (Konya)	36 (17-53)	34				
									UK (Dorset)	Greece (Xanthi)	42 (19-58)	21				

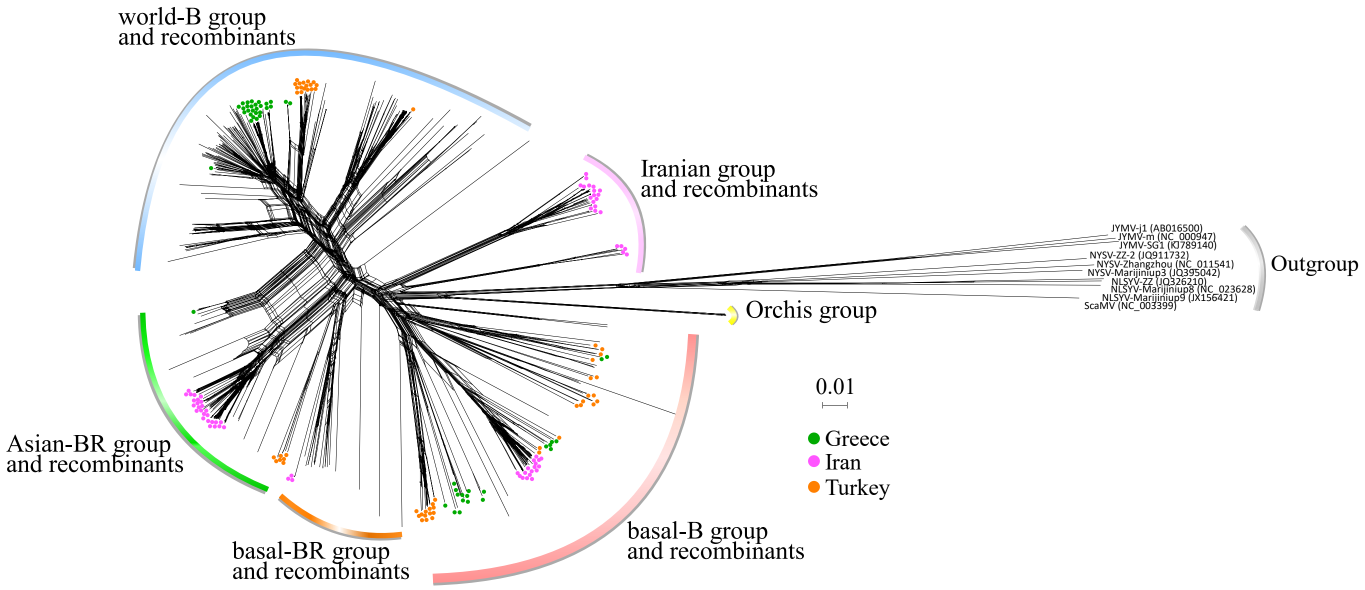

Supplementary Figure S1. A phylogenetic network of full genomic sequences of turnip mosaic virus. The network inferred from the full genomic sequences of Greece, Iran, Turkey and worldwide isolates. The genomic sequence of the isolates of narcissus late season yellows virus (NLSYV), narcissus yellow stripe virus (NYSV), Japanese yam mosaic virus (JYMV), scallion mosaic virus (ScaMV) were used as outgroup taxons because those viruses are members of turnip mosaic virus phylogenetic group. For the details of isolates, refer Table S1.

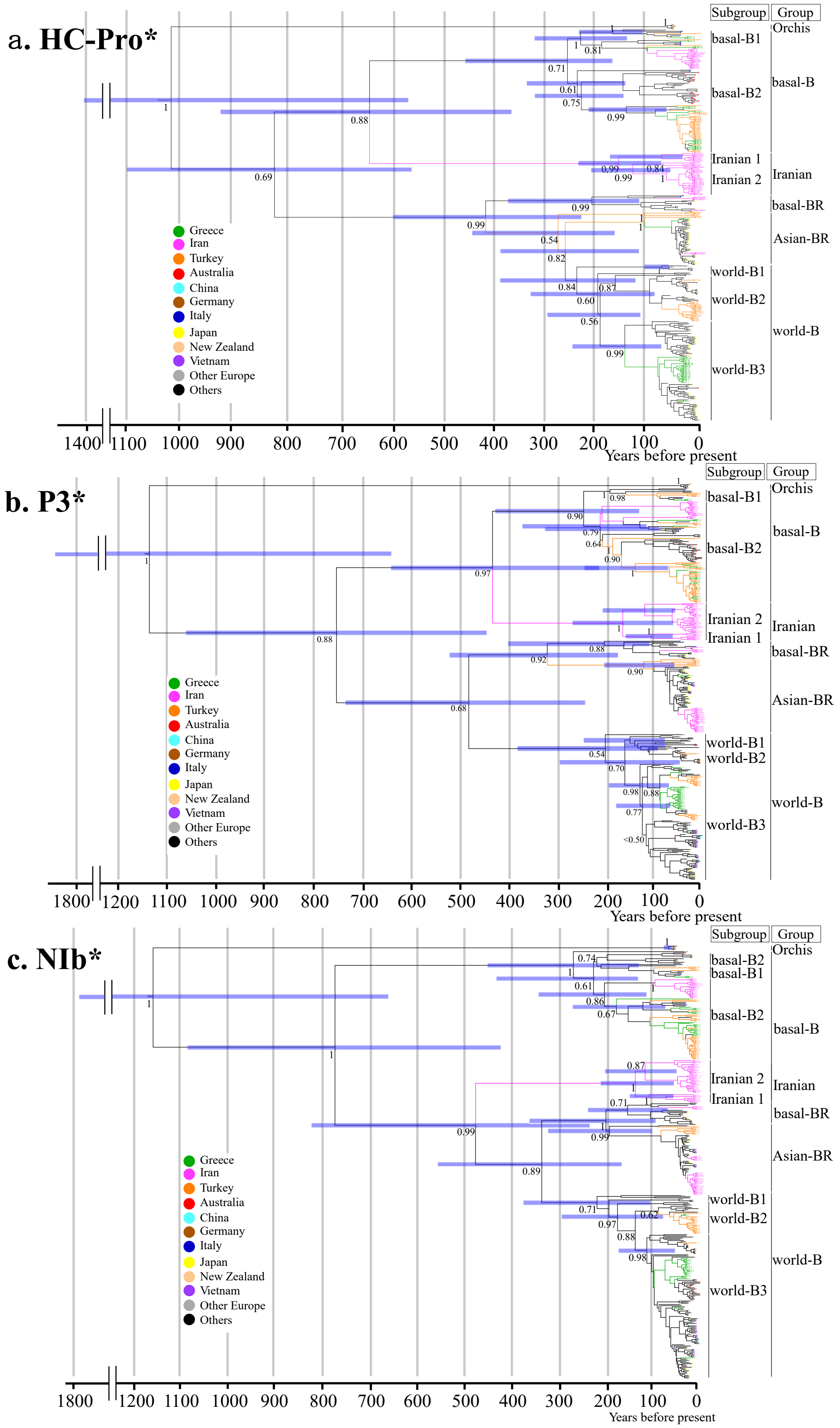

Supplementary Figure S2. Bayesian maximum-clade-credibility chronograms inferred from the polyprotein-coding regions of turnip mosaic virus genomes. The tree was estimated from the (a) HC-Pro* (partial helper-component proteinase), (b) P3* (partial protein 3) and (c) NIb* (partial nuclear inclusion b) sequences those have no recombination cross-over points. Details of the regions are given in Methods. Horizontal blue bars represent the 95% credibility intervals of estimates of node ages.

a. HC-Pro*

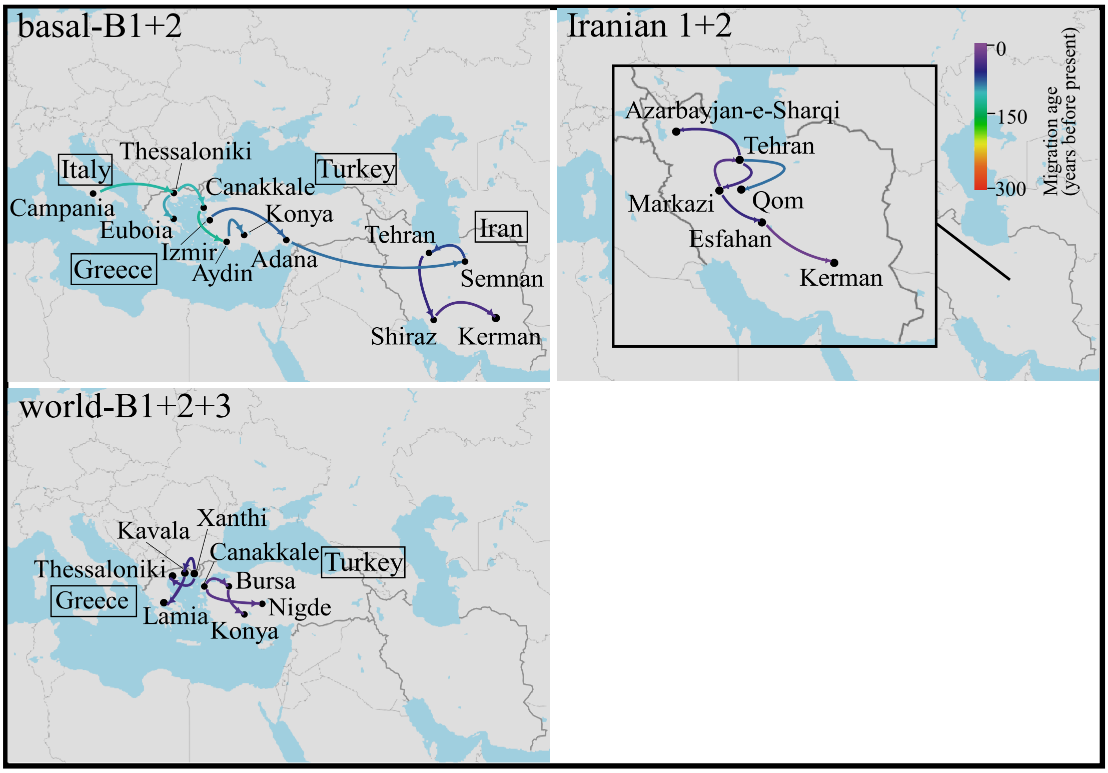

b. NIb*

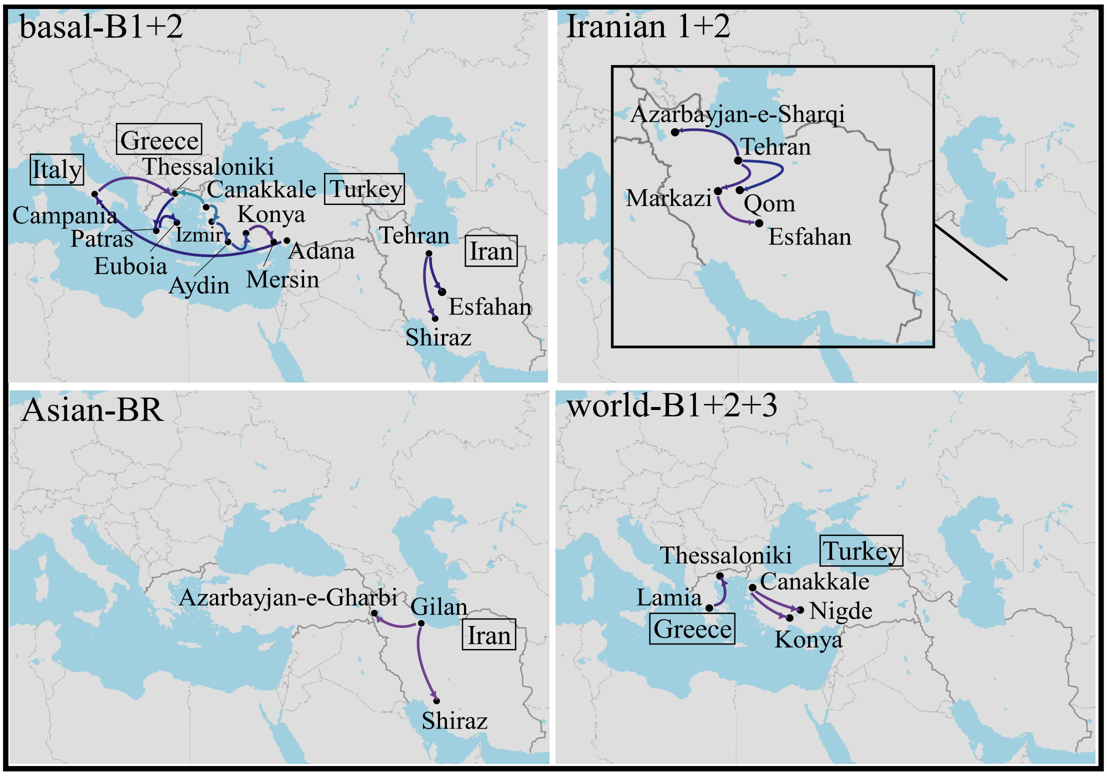

© Mapbox

Supplementary Figure S3. Plausible historical dissemination pathways of turnip mosaic virus inferred using the HC-Pro* (partial helper-component proteinase) and NIb* (partial nuclear inclusion b) sequences using those have no recombination cross-over points. Details of the regions of HC-Pro* and NIb* are given in Methods. Dissemination routes for Asia Minor and its neighboring countries are only shown, and when supported by a Bayes factor (BF) >10. Only the dissemination pathways for basal-B1+2, Iranian 1+2, Asian-BR and world-B1+2+3 group (subgroup) isolates are shown. (https://www.mapbox.com/about/maps/)

Supplementary References

1. Nguyen, H. D. *et al.* Turnip mosaic potyvirus probably first spread to Eurasian brassica crops from wild orchids about 1000 years ago. *PLOS ONE* **8**, e55336 (2013).
2. Ohshima, K. *et al.* Patterns of recombination in turnip mosaic virus genomic sequences indicate hotspots of recombination. *J Gen Virol* **88**, 298-315 (2007).
3. Farzadfar, S. *et al.* Molecular characterization of *Turnip mosaic virus* isolates from Brassicaceae weeds. *Eur J Plant Pathol* **124**, 45-55 (2009).
4. Korkmaz, S., Tomitaka, Y., Onder, S. & Ohshima, K. Occurrence and molecular characterization of Turkish isolates of *Turnip mosaic virus*. *Plant Pathol* **57**, 1155-1162 (2008).
5. Jenner, C. E., Tomimura, K., Ohshima, K., Hughes, S. L. & Walsh, J. A. Mutations in *Turnip mosaic virus* P3 and cylindrical inclusion proteins are separately required to overcome two *Brassica napus* resistance genes. *Virology* **300**, 50-59 (2002).
6. Tomimura, K., Gibbs, A. J., Jenner, C. E., Walsh, J. A. & Ohshima, K. The phylogeny of *Turnip mosaic virus*; comparisons of 38 genomic sequences reveal a Eurasian origin and a recent 'emergence' in east Asia. *Mol Ecol* **12**, 2099-2111 (2003).
7. Wang, H. Y. *et al.* Complete genomic sequence analyses of *Turnip mosaic virus* basal-BR isolates from China. *Virus Genes* **38**, 421-428 (2009).
8. Ohshima, K., Tanaka, M. & Sako, N. The complete nucleotide sequence of turnip mosaic virus RNA Japanese strain. *Arch Virol* **141**, 1991-1997 (1996).
9. Suehiro, N., Natsuaki, T., Watanabe, T. & Okuda, S. An important determinant of the ability of *Turnip mosaic virus* to infect *Brassica* spp. and/or *Raphanus sativus* is in its P3 protein. *J Gen Virol* **85**, 2087-2098 (2004).
10. Chen, C. C. *et al.* Identification of *Turnip mosaic virus* isolates causing yellow stripe and spot on calla lily. *Plant Dis* **87**, 901-905 (2003).
11. Nguyen, H. D., Tran, H. T. N. & Ohshima, K. Genetic variation of the *Turnip mosaic virus* population of Vietnam: A case study of founder, regional and local influences. *Virus Res* **171**, 138-149 (2013).
12. Kozubek, E., Irzykowski, W. & Lehmann, P. Genetic and molecular variability of a *Turnip mosaic virus* population from horseradish (*Cochlearia armoracia* L.). *J Appl Genet* **48**, 295-306 (2007).
13. Zubareva, I. A. *et al.* Genetic diversity of turnip mosaic virus and the mechanism of its transmission by Brassica seeds. *Doki Biochem Biophys* **450**, 119-122 (2013).
14. Pallett, D. W. *et al.* Variation in the pathogenicity of two *Turnip mosaic virus* isolates in wild *Brassica rapa* provenances. *Plant Pathol* **57**, 401-407 (2007).
15. Jenner, C. E. *et al.* The dual role of the potyvirus P3 protein of *Turnip mosaic virus* as a symptom and avirulence determinant in brassicas. *Mol Plant Microbe Interact* **16**, 777-784 (2003).
16. Music, M. Š. *et al.* Multilocus sequence analysis of '*Candidatus* Phytoplasma asteris' strain and the genome analysis of *Turnip mosaic virus* co-infecting oilseed rape. *J Appl Microbiol* **117**, 774-785 (2014).
17. Yasaka, R. *et al.* Phylodynamic evidence of the migration of turnip mosaic potyvirus from Europe to Australia and New Zealand. *J Gen Virol* **96**, 701-713 (2015).
18. Nyalugwe, E. P., Jones, R. A. C., Barbetti, M. J., Kehoe, M. A. Biological and molecular variation amongst Australian turnip mosaic virus isolates. *Plant Pathol* **64**, 1215-1223 (2015).
19. Nicolas, O. & Laliberté, J. F. The use of PCR for cloning of large cDNA fragments of turnip mosaic potyvirus. *J Virol Meth* **32**, 57-66 (1991).
20. Jenner, C. E. *et al.* The cylindrical inclusion gene of *Turnip mosaic virus* encodes a pathogenic determinant to the brassica resistance gene *TuRB01*. *Mol Plant Microbe Interact* **13**, 1102-1108 (2000).
21. Martin, D. P., Murrell, B., Golden, M., Khoosal, A. & Muhire, B. RDP4: Detection and analysis of recombination patterns in virus genomes. *Virus Evol* **1**, 1-5 (2015).
22. Gibbs, M. J., Armstrong, J. S. & Gibbs, A. J. Sister-scanning: a Monte Carlo procedure for assessing signals in recombinant sequences. *Bioinformatics* **16**, 573-582 (2000).
